# Construction and application of machine learning models for predicting intradialytic hypotension

**DOI:** 10.1371/journal.pone.0333357

**Published:** 2025-10-08

**Authors:** Pingping Wang, Ningjie Xu, Lingping Wu, Yue Hong, Yihui Qu, Zhijian Ren, Qun Luo, Kedan Cai

**Affiliations:** 1 Department of Nephrology, Ningbo No. 2 Hospital, Ningbo, PR China; 2 Department of Rehabilitation, Ninghai First Hospital, Ningbo, PR China; 3 Health Science center, Ningbo University, Ningbo, PR China; 4 Department of Nephrology, Ninghai Country Hospital of Traditional Chinese Medicine, Ningbo, PR China; University of Pennsylvania Perelman School of Medicine, UNITED STATES OF AMERICA

## Abstract

**Introduction:**

Intradialytic hypotension (IDH) remains a prevalent complication of hemodialysis, which is associated with adverse outcomes for patients. This study seeks to harness machine learning to construct predictive models for IDH based on multiple definitions.

**Methods:**

In this study, a comprehensive approach was employed, leveraging a dataset comprising 26,690 hemodialysis (HD) sessions for training and testing cohort, with an additional 12,293 HD sessions serving as a temporal validation cohort. Five definitions of IDH were employed, and models for each IDH definition were constructed using ten machine learning algorithms. Subsequently, model interpretation was facilitated. Feature simplification ensued, leading to the creation and evaluation of a streamlined machine learning model. Both the most effective machine learning model and its simplified counterpart underwent temporal validation.

**Results:**

Across the five distinct definitions of IDH, the CatBoost model demonstrated superior predictive prowess, generally yielding the highest receiver operating characteristic – area under the curve (ROC-AUC) (Definition 1–5: 0.859, 0.864, 0.880, 0.848, 0.845). Noteworthy is the persistent inclusion of certain features within the top 20 across all definitions, including left ventricular mass index (LVMI), etc. Leveraging these features, we developed robust machine learning models that exhibited good performance (ROC-AUC for Definition 1–5: 0.866, 0.858, 0.874, 0.843, 0.838). Both the leading original machine learning model and the refined simplified machine learning model demonstrated robust performance on a temporal validation set.

**Conclusion:**

Machine learning emerged as a reliable tool for predicting IDH in HD patients. Notably, LVMI emerged as a crucial feature for effectively predicting IDH. The simplified models are accessible on the provided website.

## 1. Introduction

Chronic Kidney Disease (CKD) presents a significant public health challenge, with a global burden of 697.5 million cases recorded in 2017 (prevalence estimate: 9.1%). Alarming is the 29.3% increase in the all-age prevalence of CKD since 1990, propelling it to become the 12th leading cause of death worldwide by 2017 [1]. The introduction of hemodialysis (HD) has played a pivotal role in extending the lives of patients at stage 5 [2]. As renal replacement therapy availability has surged, the dialysis patient population witnessed a 43.1% rise since 1990, reaching a global dialysis rate of 0.041% in 2017 [1]. Projections suggest a doubling of individuals undergoing renal replacement therapy to 5.4 million by 2030 [3]. Despite advancements in hemodialysis, the morbidity and mortality rates for treated patients remain stubbornly high [2].

Intradialytic Hypotension (IDH) stands out as one of the prevalent complications in HD. A study by Keane et al. reported a 12% frequency of IDH based on 785,682 HD sessions among 4,348 patients across 21 dialysis clinics in the USA [4]. IDH is intricately linked with adverse outcomes in HD patients, encompassing cardiovascular complications, arteriovenous fistula thrombosis, diminished residual renal function, and increased mortality [5]. Presently, the prediction of IDH relies heavily on clinical experience, lacking the support of mature clinical applications of intelligent software. Therefore, there is a critical and pressing need to develop a robust prediction model for IDH.

Medical technologies based on artificial intelligence are progressing at a rapid pace [6]. Across diverse medical domains [7–9], machine learning is emerging as a crucial and, in some cases, indispensable tool, empowering healthcare professionals to render more precise and swifter decisions [10]. Despite this, the application of machine learning methods for predicting the onset of IDH has been relatively limited in recent years. These studies encounter certain constraints. Firstly, the absence of consensus on IDH definitions has led to variations in the definitions employed across current research [11,12]. Secondly, the spectrum of clinical features gathered in these studies is diverse, encompassing blood test indices, medication profiles, hemodialysis-related parameters, co-morbidities, and cardiothoracic ratios, but none have incorporated echocardiography-related parameters [13–16]. However, this study highlights the potential of echocardiographic assessments as a predictive tool for IDH [17].

In summary, the primary objective of this study was to develop multiple machine learning models for predicting IDH in HD, utilizing various definitions of IDH that are applicable in diverse scenarios to mitigate complications in patients. Additionally, to enhance the precision of these models, we incorporated echocardiography-based clinical features, marking the advanced effort in constructing prediction models of this nature.

## 2. Methods

### 2.1. Patients

#### 2.1.1. Training and testing cohort.

A total of 36,271 hemodialysis sessions, involving patients undergoing maintenance hemodialysis at the Hemodialysis Center of Ningbo NO.2 Hospital (Zhejiang, China) from August 1, 2019, to June 30, 2022, were included in this study. Exclusion criteria comprised: (i) age < 18 years; (ii) pre-dialysis systolic blood pressure <90 mmHg; (iii) Missing data. After excluding 61 hemodialysis sessions with age < 18 years, 115 with pre-dialysis systolic blood pressure <90 mmHg, and 9,405 with missing data, a total of 26,690 hemodialysis sessions (132 patients) remained for analysis.

#### 2.1.2. Temporal validation cohort.

A total of 15,757 hemodialysis sessions conducted between July 1, 2022, and June 30, 2023, at the same Hemodialysis Center were incorporated into this study. The exclusion criteria mirrored those applied to the training and testing cohort. Subsequently, no hemodialysis sessions were excluded due to age below 18 years, 69 were excluded for pre-dialysis systolic blood pressure below 90 mmHg, and 3,395 were excluded for missing data. This resulted in 12,293 hemodialysis sessions (106 patients: 91 patients with extended follow-up data from the training and testing cohort and 15 newly recruited patients (contributing 924 HD sessions)) forming the basis of the study’s analysis.

This study underwent review and approval by the Ethics Committee of Ningbo NO.2 Hospital (Approval ID: KY202301501). The research adhered to the principles outlined in the Declaration of Helsinki. Given the retrospective nature of this study, approval was secured for a waiver of informed consent. The data were accessed for research purposes on July 10–18, 2023, and authors had no access to information that could identify individual participants during or after data collection.

### 2.2. Clinical features

A total of 44 clinical features were collected for this study, encompassing demographic characteristics (gender (male, female), age, dialysis vintage (DV), height, weight, and body mass index (BMI)), primary diseases (hypertensive nephrosclerosis (HN), diabetic nephropathy (DN), gouty nephropathy (GN), chronic glomerulonephritis (CGN), among others), comorbidities (hypertension (HTN), diabetes mellitus (DM), gout), vital signs (systolic blood pressure (SBP), diastolic blood pressure (DBP), mean arterial pressure (MAP), heart rate (HR) [18]), laboratory tests (white blood cell count (WBC) [19], hemoglobin (Hb) [20], hematocrit (Hct), platelet count (Plt), potassium (K), sodium (Na) [21], phosphorus (P) [22], calcium (Ca), albumin (Alb) [23], parathyroid hormone (PTH) [24]), ultrasound and imaging examinations (cardiothoracic ratio (CTR), left ventricular mass index (LVMI), ejection fraction (EF)), hemodialysis access (autologous arteriovenous fistula (AVF), grafted vascular fistula (GVF), tunneled cuffed catheter (TCC), non-tunneled catheter (NTC)), dialysis settings (dialysis frequency (DF), dialysis duration (DD), dry weight (DW), interdialytic weight gain rate (IDWGR), ultrafiltration volume (UFV), ultrafiltration rate (UFR)), and dialysis adequacy indexes (urea clearance index (Kt/V), urea reduction ratio (URR)). Blood pressure measurements were obtained using the COMEN electrocardiogram monitor (model STAR8000E) throughout the study period, with no changes to the measurement protocol.

The assessment frequency for each feature is presented as follows:

Variable over time: age, DV, weight, BMI, vital signs, hemodialysis access, DF, DD, DW, IDWGR, UFV, UFR.

Monthly: WBC, Hb, Hct, Plt.

Quarterly: K, Na, P, Ca, Alb, PTH, Kt/V, URR.

Annually: height, comorbidities, CTR, LVMI, EF.

Fixed: gender, primary diseases.

In this study, the most recent values of each feature were selected for every hemodialysis session.

### 2.3. Data preprocessing

#### 2.3.1. Categorical features processing.

The categorical attributes within the training and testing cohort, as well as the temporal validation cohort, encompassed gender, primary diseases, comorbidities, and hemodialysis access. To facilitate their integration into the analysis, all these categorical features underwent transformation into one-hot encodings.

#### 2.3.2. Labeling of target values.

In adherence to established clinical guidelines and insights derived from clinical studies, this study adopts five distinct definitions for IDH, outlined as follows: (i) Definition 1: systolic blood pressure <90 mmHg during dialysis [13,25,26]; (ii) Definition 2: systolic blood pressure exhibiting a decrease of ≥20 mmHg during dialysis compared to pre-dialysis levels [26–28]; (iii) Definition 3: systolic blood pressure showing a reduction of ≥30 mmHg during dialysis compared to pre-dialysis levels [13]; (iv) Definition 4: systolic blood pressure demonstrating a decrease of ≥20 mmHg during dialysis compared to pre-dialysis levels and/or mean arterial pressure falling by ≥10 mmHg during dialysis compared to pre-dialysis levels [12,25,26,29,30]; (v) Definition 5: systolic blood pressure registering a decline of ≥30 mmHg during dialysis compared to pre-dialysis levels and/or mean arterial pressure dropping by ≥10 mmHg during dialysis compared to pre-dialysis levels [31].

The hemodialysis sessions were labeled according to each of the above 5 definitions in the training and testing cohort and the temporal validation cohort, with the absence of IDH labeled as “0” and the presence of IDH labeled as “1”.

### 2.4. Model construction

In this research, Python 3.13.5 (Python Software Foundation, Beaverton, USA) served as the primary platform for conducting model training, testing, validation, and application. The essential Python packages employed encompassed scikit-learn (version 1.6.1), numpy (version 2.1.3), pandas (version 2.2.3), matplotlib (version 3.10.0), imblearn (0.12.2), xgboost (version 2.1.1), lightgbm (version 4.6.0), catboost (version 1.2.8), scipy (version 1.15.3), joblib (version 1.4.2), shap (version 0.48.0), streamlit (version 1.45.1), among others. The training and testing cohort was randomly partitioned into a training set comprising 75% of the data and a testing set comprising 25%, utilizing the “train_test_split” method from the “sklearn.model_selection” module. Subsequently, the feature data within the training set underwent normalization via the “MinMaxScaler” method from the “sklearn.preprocessing” module, and the normalization of feature data in the testing set was performed based on the transformation fitted to the training set’s feature data. The synthetic minority over-sampling technique (SMOTE) was applied to address class imbalance in the training set.

We selected 10 machine learning algorithms for the purpose of this study. The chosen algorithms are k-nearest neighbor (KNN), logistic regression (LR), decision tree (DT), extremely randomized tree (ET), random forest (RF), gradient boosting decision tree (GBDT), light gradient boosting machine (LGBM), extreme gradient boosting (XGBoost), CatBoost, and adaptive boosting (AdaBoost). These algorithms have been specifically curated to facilitate model training across the five distinct definitions of IDH employed in our research.

### 2.5. Model evaluation and interpretation

In this study, model performance was evaluated using accuracy, precision, recall, F1-score, receiver operating characteristic – area under the curve (ROC-AUC), and precision recall – area under the curve (PR-AUC). ROC curves and PR curves were also plotted. A comparative analysis was conducted across 10 distinct machine learning algorithm models, evaluating their performance across the five definitions of IDH. Subsequently, the optimal machine learning algorithm model for each IDH definition was identified based on the collective evaluation criteria.

The features of the most effective machine learning algorithm models for the five IDH definitions were ranked in descending order of importance, employing shapley additive explanation (SHAP) for comprehensive model interpretation.

### 2.6. Feature simplification and re-modeling

The most important features ranked in the top 5, 10, 15, and 20 for each of the five IDH definitions were included in the study. The best machine learning algorithms were employed to create simpler models for each definition. The performance of these simplified machine learning models was evaluated using accuracy, precision, recall, F1-score, ROC-AUC, and PR-AUC, and corresponding curves were plotted.

### 2.7. Temporal validation

The dataset of the temporal validation cohort after data preprocessing served as the temporal validation set. To ensure consistency, the feature data in the temporal validation set underwent normalization based on the fitted transformation derived from the feature data in the training set. Both the best machine learning model and the optimal simplified machine learning models for each of the five IDH definitions underwent temporal validation, aiming to provide a comprehensive evaluation of the performance of both the original and simplified models.

### 2.8. Statistical analysis

All statistical analyses were conducted using SPSS software, version 24.0 (SPSS Inc., Chicago, IL, USA). Continuous variables were presented as means with standard deviation (SD), while categorical variables were expressed in terms of frequencies and percentages.

## 3. Results

In this study, a total of 26,690 hemodialysis sessions were included in the training and testing cohort, while the temporal validation cohort comprised 12,293 hemodialysis sessions. The incidences of IDH, based on Definition 1–5, were 4.88%, 46.71%, 27.71%, 55.25%, and 48.81%, respectively, in the training and testing cohort. In the temporal validation cohort, the corresponding incidences were 3.89%,45.95%, 27.16%, 54.51%, and 48.11%. Notably, the incidence of IDH for Definition 1 and 3 was lower than the other definitions in both cohorts. To address this class imbalance, we applied SMOTE exclusively to training sets for Definition 1 and 3.

### 3.1. Baseline characteristics

Table 1 presents 44 baseline characteristics, including demographic details, primary diseases, comorbidities, vital signs, laboratory tests, ultrasound and imaging examinations, hemodialysis access and settings, as well as dialysis adequacy indexes. No differences were observed in age and gender between groups: in the training and testing cohort, the male proportion was 66.92%, with an average age of 61.19 ± 14.18 years, and in the temporal validation cohort, the male proportion was 65.26%, with an average age of 61.88 ± 13.5 years. Chronic glomerulonephritis constituted the largest proportion of primary diseases in both cohorts (58.70% and 67.58%, respectively), while hypertension emerged as the predominant comorbidity in both groups (76.65% and 70.67%, respectively).

**Table 1:** Baseline characteristics.

	Training and testing cohort (n = 26690)	Temporal validation cohort (n = 12293)
Demographic characteristics		
Male (n;%)	17861 (66.92%)	8022 (65.26%)
Female (n;%)	8829 (33.08%)	4271 (34.74%)
Age (year)	61.19 ± 14.18	61.88 ± 13.50
DV (dialysis vintage) (year)	3.36 ± 2.74	4.32 ± 3.09
Height (cm)	165.69 ± 8.74	165.82 ± 8.57
Weight (kg)	62.79 ± 13.01	62.73 ± 12.77
BMI (kg/m^2^)	22.75 ± 3.59	22.71 ± 3.71
Primary diseases		
HN (hypertensive nephrosclerosis) (n;%)	374 (1.40%)	155 (1.26%)
DN (diabetic nephropathy) (n;%)	2237 (8.38%)	1220 (9.92%)
GN (gouty nephropathy) (n;%)	121 (0.45%)	0 (0.00%)
CGN (chronic glomerulonephritis) (n;%)	15666 (58.70%)	8307 (67.58%)
Others (n;%)	10187 (38.17%)	3504 (28.50%)
Comorbidities		
HTN (hypertension) (n;%)	20459 (76.65%)	8687 (70.67%)
DM (diabetes mellitus) (n;%)	3807 (14.26%)	2295 (18.67%)
Gout (n;%)	1913 (7.17%)	845 (6.87%)
Vital signs		
SBP (systolic blood pressure) (mmHg)	144.21 ± 22.48	143.84 ± 21.64
DBP (diastolic blood pressure) (mmHg)	75.02 ± 13.36	74.53 ± 12.67
MAP (mean arterial pressure) (mmHg)	98.08 ± 13.72	97.63 ± 12.92
HR (heart rate) (bpm)	78.16 ± 13.06	78.36 ± 13.04
Laboratory tests		
WBC (white blood cell) (*10^9/L)	6.16 ± 1.97	6.09 ± 2.00
Hb (hemoglobin) (g/L)	108.76 ± 16.30	110.56 ± 16.46
Hct (hematocrit) (%)	33.30 ± 5.06	33.97 ± 5.16
Plt (platelet) (*10^9/L)	180.51 ± 53.28	186.88 ± 57.04
K (potassium) (mmol/L)	4.78 ± 0.77	4.26 ± 0.77
Na (sodium) (mmol/L)	138.78 ± 3.28	139.53 ± 2.84
P (phosphorus) (mmol/L)	1.80 ± 0.57	1.38 ± 0.49
Ca (calcium) (mmol/L)	2.25 ± 0.23	2.37 ± 0.21
Alb (albumin) (g/L)	38.28 ± 4.45	38.91 ± 4.58
PTH (parathyroid hormone) (pg/ml)	327.44 ± 263.23	267.87 ± 195.76
Ultrasound and imaging examinations		
CTR (cardiothoracic ratio)	0.49 ± 0.05	0.47 ± 0.03
LVMI (left ventricular mass index) (g/m^2^)	111.89 ± 35.06	107.03 ± 31.69
EF (ejection fraction) (%)	63.08 ± 8.89	64.68 ± 7.16
Hemodialysis access		
AVF (autologous arteriovenous fistula) (n;%)	23518 (88.12%)	11020 (89.64%)
GVF (grafted vascular fistula) (n;%)	1224 (4.59%)	984 (8.00%)
TCC (Tunneled Cuffed Catheter) (n;%)	1726 (6.47%)	80 (0.65%)
NTC (Non-tunneled Catheter) (n;%)	222 (0.83%)	209 (1.70%)
Dialysis settings		
DF (dialysis frequency) (per week)	2.81 ± 0.53	2.82 ± 0.50
DD (dialysis duration) (h)	3.97 ± 0.15	3.98 ± 0.12
DW (dry weight) (kg)	60.10 ± 12.70	60.10 ± 12.47
IDWGR (interdialytic weight gain rate)	0.04 ± 0.02	0.04 ± 0.02
UFV (ultrafiltration volume) (L)	2.64 ± 1.03	2.65 ± 0.92
UFR (ultrafiltration rate) (%)	0.67 ± 0.26	0.67 ± 0.24
Dialysis adequacy indexes		
Kt/V (urea clearance index)	1.57 ± 0.63	1.50 ± 0.30
URR (urea reduction ratio)	0.71 ± 0.09	0.71 ± 0.06
Target variable		
Defn1 (definition 1) (n;%)	1302 (4.88%)	478 (3.89%)
Defn2 (definition 2) (n;%)	12466 (46.71%)	5649 (45.95%)
Defn3 (definition 3) (n;%)	7396 (27.71%)	3339 (27.16%)
Defn4 (definition 4) (n;%)	14746 (55.25%)	6701 (54.51%)
Defn5 (definition 5) (n;%)	13027 (48.81%)	5914 (48.11%)

Results are shown for the baseline characteristics of the training and testing cohorts together, vs temporal validation cohort. There were 26,690 HD sessions for the training and testing cohort and 12,293 HD sessions for the temporal validation cohort. Continuous variables are expressed as mean ± standard deviation and categorical variables as number (percentage (%)). ‘Defn1’, ‘Defn2’, ‘Defn3’, ‘Defn4’, and ‘Defn5’ represent the 5 definitions of IDH, respectively.

### 3.2. Model evaluation

**Fig 1:**
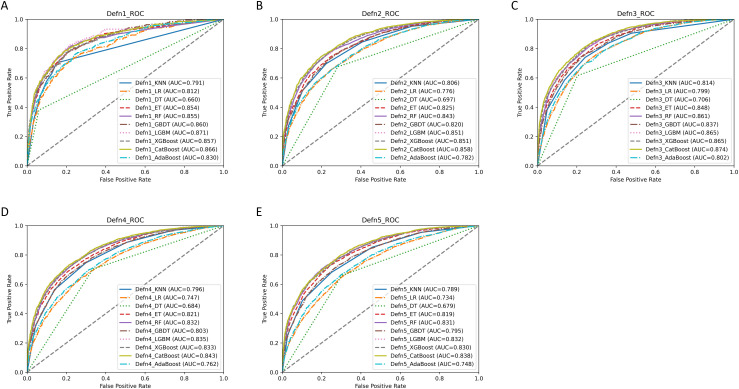
ROC curves of 10 machine learning models for the 5 definitions of IDH. A–E show the ROC curves and ROC-AUC of 10 machine learning models for the 5 definitions of IDH, respectively. ‘Defn1’, ‘Defn2’, ‘Defn3’, ‘Defn4’, and ‘Defn5’ represent the 5 definitions of IDH, respectively. KNN, k-nearest neighbor; LR, Logistic Regression; DT, Decision Tree; ET, Extremely randomized Tree; RF, Random Forest; GBDT, Gradient Boosting Decision Tree; LGBM, Light Gradient Boosting Machine; XGBoost, Extreme Gradient Boosting; AdaBoost, Adaptive Boosting. ROC, Receiver Operating Characteristic Curve; AUC, Area Under Curve.

S1 Fig illustrates the accuracy, precision, recall, F1-score, and ROC-AUC (with corresponding 95% confidence intervals (CI) presented in S1 Table) and PR-AUC of the 10 machine learning models across the five definitions of IDH. The ROC plots are provided in Fig 1, while the PR plots are available in S2 Fig. The findings revealed that, across all five definitions of IDH, the CatBoost model generally achieved the highest ROC-AUC and PR-AUC values (ROC-AUC of CatBoost model for Definition 1–5: 0.866 (0.841–0.891), 0.858 (0.849–0.867), 0.874 (0.863–0.885), 0.843 (0.833–0.852), 0.838 (0.828–0.847)). Moreover, its accuracy, precision, recall, and F1 scores were notably ranked high. Consequently, the CatBoost model emerged as the optimal machine learning model for all definitions of IDH.

### 3.3. Model interpretation

**Fig 2:**
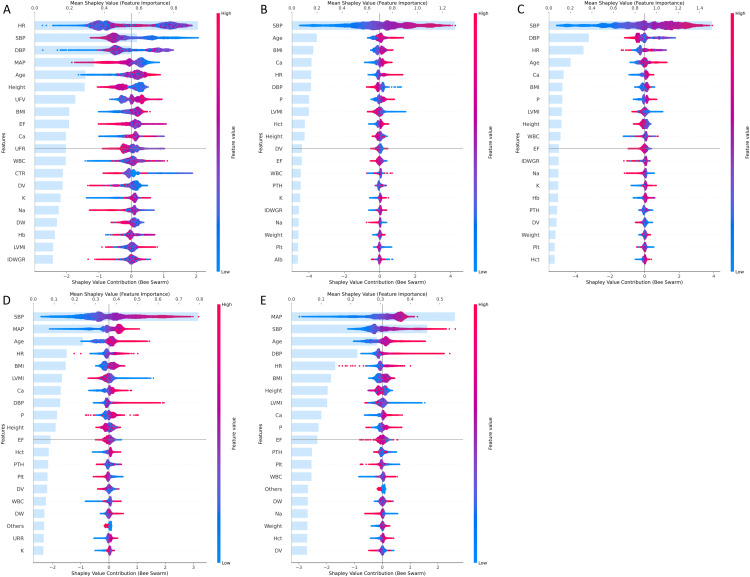
SHAP summary plots of CatBoost models for the 5 definitions of IDH. A–E show the SHAP summary plots of CatBoost models for the 5 definitions of IDH, respectively. The top 20 features in order of importance are shown in the plots, and the horizontal coordinates in the plots are the SHAP values. A dot is created for the SHAP value of each feature for each HD session, with red indicating high feature values and blue indicating low feature values. The more positive a point’s SHAP value is, the more positively it affects the prediction, and conversely, the more negative a point’s SHAP value is, the more negatively it affects the prediction.

The SHAP summary plots of the CatBoost models for the five IDH definitions are displayed in Fig 2, where red signifies high values, blue indicates low values, and the left side of the “0” axis indicates a negative impact on positive prediction result, while the right side of the “0” axis indicates a positive effect on positive prediction result. As shown in Fig 2, low LVMI was identified as a risk factor for IDH across Definition 2–5, while showed no consistent directional association for Definition 1.

We also condensed the feature importance summary, presented in S2 Table, where “√√√√”, “√√√”, “√√”, “√” denote features that ranked in the top 5, top 10, top 15, and top 20 in importance across all IDH definitions, respectively. The results unveiled that features consistently within the top 5 were age, SBP, and HR; those within the top 10 were height, BMI, DBP, and Ca; the top 15 contained EF; and the top 20 comprised DV, WBC, and LVMI. Subsequently, we utilized all features within the top 5 (3 features: age, SBP, and HR), top 10 (7 features: age, SBP, HR, height, BMI, DBP, and Ca), top 15 (8 features: age, SBP, HR, height, BMI, DBP, Ca, and EF), and top 20 (11 features: age, SBP, HR, height, BMI, DBP, Ca, EF, DV, WBC, and LVMI) to construct streamlined machine learning models for each IDH definition. Sensitivity analysis for LVMI (S3 Fig) showed noticeable separation among multiple cumulative distribution function (CDF) curves across Definition 2–5, indicating that the model output is sensitive to LVMI. However, for Definition 1, the CDF curves showed limited separation, suggesting lower sensitivity of the model output to LVMI. These results are consistent with the SHAP analysis.

To assess the differential predictive value of LVMI for IDH across varying pre-dialysis SBP ranges, we stratified patients into two subgroups: 90 mmHg ≤ SBP < 130 mmHg subgroup and SBP ≥ 130 mmHg subgroup. As demonstrated in S4–S5 Figs and S3 Table, the subgroup with SBP ≥ 130 mmHg exhibited higher SHAP value and superior importance rank for LVMI, suggesting that LVMI may play a more substantial role in predicting IDH among patients with elevated pre-dialysis SBP.

### 3.4. Evaluation of the simplified version of the model

**Fig 3:**
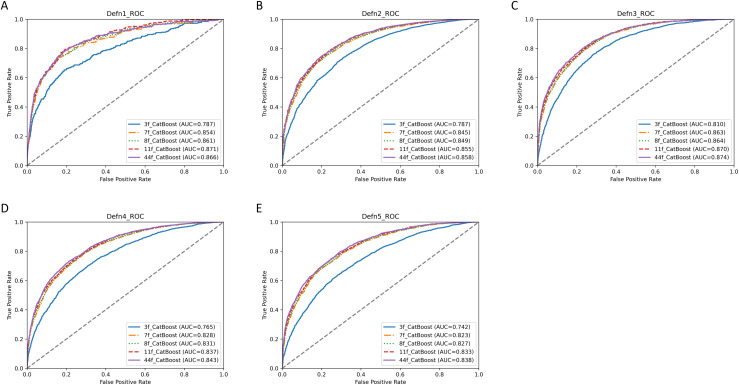
ROC curves of the simplified CatBoost models for the 5 definitions of IDH. A–E show the ROC curves and ROC-AUC of the simplified CatBoost models for the 5 definitions of IDH, respectively. ‘Defn1’, ‘Defn2’, ‘Defn3’, ‘Defn4’, and ‘Defn5’ represent the 5 definitions of IDH, respectively. ‘3f’, ‘7f’, ‘8f’, and ‘11f’ represent the simplified CatBoost models with 3 features, 7 features, 8 features, and 11 features, respectively. ‘44f’ represents the original CatBoost model (with 44 features). ROC, Receiver Operating Characteristic Curve; AUC, Area Under Curve.

Similarly, we assessed the simplified machine learning model, presenting its accuracy, precision, recall, F1 score, ROC-AUC (with corresponding 95% CI detailed in S4 Table), and PR-AUC for 3, 7, 8, 11, and 44 (all) features across the five IDH definitions in S6 Fig. The ROC plot is depicted in Fig 3, and the PR plot is shown in S7 Fig. The findings revealed that the ROC-AUC, PR-AUC, accuracy, precision, recall, and F1 score of the machine learning model with 11 features closely mirrored those of the model with 44 features across all five IDH definitions (ROC-AUC of the machine learning model for 11 features across Definition 1–5: 0.871 (0.846–0.896), 0.855 (0.846–0.864), 0.870 (0.859–0.881), 0.837 (0.828–0.846), 0.833 (0.823–0.843)). Hence, the results indicated that the machine learning model with 11 features stood out as the optimal simplified model across all five IDH definitions.

### 3.5. Temporal validation

**Fig 4:**
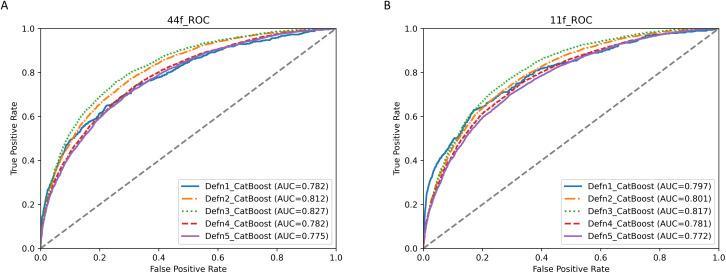
Model validation (ROC curves) of the original CatBoost models and the simplified CatBoost models for the 5 definitions of IDH. A and B show the model validation (ROC curves and ROC-AUC) results of the original CatBoost models and the simplified CatBoost models for the 5 definitions of IDH, respectively. ‘Defn1’, ‘Defn2’, ‘Defn3’, ‘Defn4’, and ‘Defn5’ represent the 5 definitions of IDH, respectively. ‘11f’ represents the simplified CatBoost model (with 11 features), and ‘44f’ represents the original CatBoost model (with 44 features). ROC, Receiver Operating Characteristic Curve; AUC, Area Under Curve.

We conducted temporal validation of both the best original machine learning model and the best simplified machine learning model for the five IDH definitions. Evaluation metrics, including accuracy, precision, recall, F1 score, ROC-AUC (with corresponding 95% CI detailed in S5 Table), and PR-AUC, are depicted in S8 Fig. The ROC plots are presented in Fig 4, and the PR plots are available in S9 Fig. The results demonstrated that both the best original machine learning model and the best simplified machine learning model exhibited robust performance on the temporal validation set (ROC-AUC: 0.782 (0.757–0.807), 0.812 (0.804–0.820), 0.827 (0.818–0.836), 0.782 (0.774–0.790), 0.775 (0.767–0.784) for the best original machine learning models across Definition 1–5; ROC-AUC: 0.797 (0.773–0.821), 0.801 (0.793–0.809), 0.817 (0.807–0.826), 0.781 (0.773–0.789), 0.772 (0.764–0.780) for the best simplified machine learning models across Definition 1–5).

Since 85.85% of the patients in the temporal validation set had extended follow-up data overlapping with the training and testing cohort, an additional validation was performed using 15 newly recruited patients,who contributed 924 HD sessions including 56 positive outcomes (ROC-AUC: 0.564 (0.484–0.645), 0.682 (0.646–0.718), 0.692 (0.647–0.736), 0.661 (0.626–0.696), 0.623 (0.586–0.659) for the best original machine learning models across Definition 1–5; ROC-AUC: 0.716 (0.638–0.794), 0.680 (0.643–0.716), 0.703 (0.659–0.747), 0.663 (0.628–0.697), 0.633 (0.597–0.669) for the best simplified machine learning models across Definition 1–5). The corresponding ROC curves are presented in S10 Fig.

### 3.6. Models application

**Fig 5:**
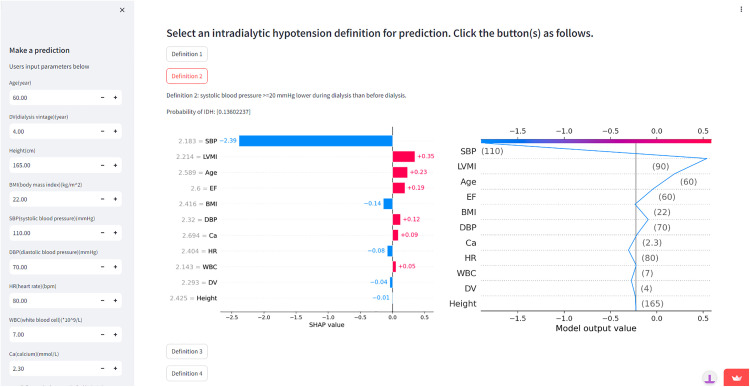
Input-Output Demonstration of the IDH Prediction System.

To enhance user-friendliness for individuals utilizing the simplified machine learning models for IDH prediction, we have designed an IDH prediction system, as illustrated in Fig 5. Additionally, to examine the overlapping categories across the five definitions, we labeled HD sessions meeting any of the five IDH definitions as ‘1’. We constructed a composite model incorporating the overlapping definitions (including 11 key features and utilizing the CatBoost algorithm), which chieved an ROC-AUC of 0.835 (95% CI: 0.826–0.845). Additional model evaluation results are presented in S6 Table, while the ROC curve and PR curve are shown in S11 Fig. This integrated model was subsequently integrated into our IDH prediction system, and can be accessed by selecting the ‘Overlapping Definitions’ option, which outputs comprehensive prediction probabilities. This system can be accessed through the following website: https://idhpred.streamlit.app/.

We simulated a standardized patient with the following characteristics: 60 years old, 4-year dialysis vintage, height 165 cm, BMI 22 kg/m^2^, pre-dialysis blood pressure 110/70 mmHg, HR 80 beats/min, WBC 7 × 10^9^/L, Ca 2.3 mmol/L, LVMI 90 g/m^2^, and EF 60%. Upon inputting these values into the IDH prediction system, the results indicated that the patient’s IDH occurrence rates were 0.208, 0.136, 0.045, 0.310, 0.291, and 0.191 for Definition 1–5 and overlapping definitions, respectively (S12 Fig).

## 4. Discussion

We developed machine learning models for predicting IDH in HD patients across five IDH definitions. Our comprehensive approach included model construction, evaluation, interpretation, and feature simplification based on feature importance analysis. Additionally, we created simplified IDH prediction machine learning models, conducted temporal validation, and successfully implemented these models.

This study made significant contributions on multiple fronts. Firstly, it introduced LVMI and EF as key features for predicting IDH, two echocardiographic indices that hadn’t been collectively employed in previous machine learning-based studies. Ngankem et al. [32], in a comparative study of IDH definitions linked to increased mortality, emphasized the enduring risk posed by heart disease, highlighting its consistent association with heightened IDH risk during treatments. Results from a retrospective cohort study further supported these findings, demonstrating a reduction in EF and an increase in left atrial diameter index (LADI) and LVMI at the end of follow-up in the IDH group (p < .05) [33]. To our knowledge, our study represents the first incorporation of these two crucial features in predicting IDH using machine learning methods.

Secondly, we employed five definitions of IDH. Given the variations in IDH definitions across guidelines and research studies, it’s noteworthy that most researchers typically focus on 1–3 definitions of IDH [25,26]. In this study, we took a comprehensive approach by incorporating up to 5 commonly used definitions of IDH for prediction modeling, aligning with the standards endorsed by various guidelines and researchers. The importance of each feature varied across different IDH definitions because Definition 1 focused on the absolute value of blood pressure, while Definition 2–5 emphasized the decrease in blood pressure. For instance, higher SBP had a negative effect on the positive prediction result for Definition 1 but a positive effect for Definition 2–5. Since Definition 1 was defined as SBP < 90 mmHg during dialysis, a higher pre-dialysis SBP generally indicated a lower likelihood of SBP falling below 90 mmHg during hemodialysis. In contrast, Definition 2–5 were based on the magnitude of decrease in SBP or MAP. Consequently, a higher pre-dialysis SBP was generally associated with a greater probability of a larger absolute decline in SBP or MAP during dialysis. For example, a rapid reduction in SBP from 180 mmHg to 150 mmHg was more likely to occur than a decrease from 100 mmHg to 70 mmHg during hemodialysis. Therefore, depending on the chosen definition of IDH, the predicted outcomes may vary. The models developed for each definition were designed for practical clinical applications, allowing users to tailor predictions according to their chosen IDH definitions or complement each other effectively for comprehensive risk prediction.

Thirdly, this study employed a diverse set of up to 10 machine learning algorithms to construct models, including the CatBoost algorithm, among others. The CatBoost algorithm, introduced in 2018, belongs to the GBDT family and has found applications in diverse fields such as astronomy, finance, medicine, and biology. Particularly well-suited for tasks involving categorical and heterogeneous data [34], CatBoost emerged as the top-performing machine learning algorithm in our study across all IDH definitions. In prior studies on machine learning-based IDH prediction, Daqing Hong et al. [15] utilized 18 machine learning algorithms, with the top three being random forest (AUC = 0.812, 95% CI = 0.811–0.813), gradient boosting (AUC = 0.748, 95% CI = 0.747–0.749), and logistic regression (AUC = 0.743, 95% CI = 0.742–0.744). Notably, our study incorporated the CatBoost algorithm, which was absent in their analysis. Jingjing Dong et al. [16] identified the LightGBM model as both interpretable and high-performing (AUC = 0.68–0.90), while Hanjie Zhang et al. [30] utilized the XGBoost algorithm, achieving an AUROC of 0.887 (95% CI = 0.881–0.892). Comparatively, Juan A Gómez-Pulido et al. [31] compared DT and Support Vector Machines (SVM), reporting slightly better accuracy for DT (75% to 81%). Jiun-Chi Huang et al. [13] demonstrated comparable prediction performance between RF and XGBoost on the training set, with RF outperforming on the testing set (R^2^ = 0.49, root mean square error (RMSE) = 16.24, mean absolute error (MAE) = 12.14). Importantly, our study incorporated the most effective algorithms from these previous studies and introduced the CatBoost algorithm as the best-performing model, representing a novel addition to the existing literature.

Fourthly, this study employed SHAP to demystify the black box of machine learning. On a global scale, the SHAP summary plot (Fig 2) unveils the importance ranking of features and their impact on predictions. Features ranking in the top 5, top 10, top 15, and top 20 for all IDH definitions were (age, SBP, HR), (height, BMI, DBP, Ca), (EF), and (DV, WBC, LVMI), respectively.Notably, Daqing Hong et al. [15] identified pre-dialysis SBP, pre-dialysis DBP, HR, dry weight, and ultrafiltration capacity as crucial factors affecting machine learning prediction accuracy. Similarly, Hanjie Zhang et al. [35] highlighted the significance of the most recent intradialytic SBP, IDH rate, and mean nadir SBP of the previous 10 dialysis sessions as top IDH predictors. In another study, Manabu Yoshimura et al. [36] developed a machine learning model to predict postinduction hypotension risk using preoperative echocardiographic data, indicating that a low LVMI value is a risk factor for postinduction hypotension. The alignment of these important features with our study suggests the credibility of our findings. In this study, SHAP analysis and sensitivity analysis revealed that higher LVMI may serve as a protective factor against IDH across Definition 2–5, but no clear directional association was observed for Definition 1. Additionally, higher SHAP values were observed in the subgroup with pre-dialysis SBP ≥ 130 mmHg, indicating a more pronounced role of LVMI in this population. These findings may be attributed to cardiac structural adaptations (myocardial hypertrophy), which contribute to a more gradual blood pressure decline and help maintain cardiac output and vital organ perfusion in the short term. However, to the best of our knowledge, definitive supporting evidence from existing literature has not yet been identified.

Fifthly, to seamlessly integrate these models into clinical applications, striking a balance between convenience and performance was crucial. Through feature simplification, we identified the optimal number of features at 11, ensuring a harmonious blend of simplicity and robust performance. Leveraging these 11 features, we constructed machine learning models for each IDH definition and conducted temporal validation on both the original and simplified models to assess their performance and generalization ability. The results demonstrate that both prediction models constructed using the CatBoost algorithm exhibit reliable discriminative performance, with the ROC AUC exceeding 0.77 on the temporal validation set. However, validation results obtained from the 15 newly recruited patients, who collectively contributed 924 hemodialysis sessions including 56 positive outcomes, showed lower ROC-AUC values compared to those derived from the entire temporal validation cohort. This discrepancy can be attributed to two main reasons. First, the small sample size and low proportion of positive outcomes in the new patient group may adversely affect the validation performance. Second, the high degree of patient overlap within the temporal validation set may lead to an overestimation of the model’s performance.

Sixthly, we have operationalized the simplified machine learning model for practical use, accessible to all users via a dedicated website. By inputting values for the 11 features (with attached calculators for BMI and LVMI), users can click the button corresponding to the selected IDH definition to obtain the probability of IDH. Additionally, the application generates SHAP plots to facilitate the interpretation of results. This interactive tool not only allows users to predict IDH probabilities for various definitions but also empowers them to identify key factors contributing to IDH. By making adjustments based on these factors, it becomes possible to mitigate the probability of IDH.

This study has certain limitations. Firstly, we did not conduct a more exhaustive examination of features. For instance, Rogerio da Hora Passos et al. [37] demonstrated that the absence of hypervolemia, as assessed by lung and vena cava ultrasound, predisposes individuals to IDH. However, this index was not included in our study. Secondly, the assessment of IDH in our study lacked the inclusion of clinical symptoms due to the unavailability of valid clinical symptom data. Notably, a limited number of studies incorporated clinical symptoms [15,28], and the inclusion of clinical symptoms, as per guidelines, is considered more comprehensive [12,29,30]. Thirdly, hyperparameters were not further optimized in this study, potentially impacting the model’s overall performance. Lastly, the temporal validation cohort and the training and testing cohort were derived from two different periods but within the same hemodialysis center, featuring some duplicated patients. This aspect may introduce potential bias in the temporal validation results.

## 5. Conclusion

In conclusion, machine learning stood as a dependable tool for predicting IDH in hemodialysis patients, with the CatBoost algorithm demonstrating superior predictive performance across all five IDH definitions. This excellence extended to both the testing set and the temporal validation set. Notably, LVMI emerged as a crucial and previously underemphasized feature for IDH prediction. The implementation of simplified models, accessible through a user-friendly website, facilitates convenient utilization by individuals.

## Supporting information

S1 Fig Model evaluation of 10 machine learning algorithms for the 5 definitions of IDH. A–E show the ROC-AUC, PR-AUC, accuracy, precision, recall, and f1-score of the 10 machine learning algorithms for the 5 definitions of IDH, respectively. ‘Defn1’, ‘Defn2’, ‘Defn3’, ‘Defn4’, and ‘Defn5’ represent the 5 definitions of IDH, respectively. KNN, k-nearest neighbor; LR, Logistic Regression; DT, Decision Tree; ET, Extremely randomized Tree; RF, Random Forest; GBDT, Gradient Boosting Decision Tree; LGBM, Light Gradient Boosting Machine; XGBoost, Extreme Gradient Boosting; AdaBoost, Adaptive Boosting; ROC, Receiver Operating Characteristic Curve; PR, Precision-Recall Curve; AUC, Area Under Curve.
(TIF)

S2 Fig PR curves of 10 machine learning models for the 5 definitions of IDH. A–E show the PR curves and PR-AUC of 10 machine learning models for the 5 definitions of IDH, respectively. ‘Defn1’, ‘Defn2’, ‘Defn3’, ‘Defn4’, and ‘Defn5’ represent the 5 definitions of IDH, respectively. KNN, k-nearest neighbor; LR, Logistic Regression; DT, Decision Tree; ET, Extremely randomized Tree; RF, Random Forest; GBDT, Gradient Boosting Decision Tree; LGBM, Light Gradient Boosting Machine; XGBoost, Extreme Gradient Boosting; AdaBoost, Adaptive Boosting; PR, Precision-Recall Curve; AUC, Area Under Curve.
(TIF)

S3 Fig Sensitivity analysis of the CatBoost model for LVMI. A–E show the CDF curves of the CatBoost model for the 5 definitions of IDH, respectively, with LVMI fixed at one of ten representative values uniformly sampled between its maximum and minimum observed values. CDF, Cumulative Distribution Function; LVMI, Left Ventricular Mass Index.
(TIF)

S4 Fig SHAP summary plots of CatBoost models for the 5 definitions of IDH (90 mmHg ≤ SBP < 130 mmHg subgroup). A–E show the SHAP summary plots of CatBoost models for the 5 definitions of IDH in this subgroup, respectively. All features are presented in descending order of importance, with their corresponding SHAP values displayed along the horizontal axis.
(TIF)

S5 Fig SHAP summary plots of CatBoost models for the 5 definitions of IDH (SBP ≥ 130 mmHg subgroup). A–E show the SHAP summary plots of CatBoost models for the 5 definitions of IDH in this subgroup, respectively. All features are presented in descending order of importance, with their corresponding SHAP values displayed along the horizontal axis.
(TIF)

S6 Fig Model evaluation of the simplified CatBoost models for the 5 definitions of IDH. A–E show the ROC-AUC, PR-AUC, accuracy, precision, recall, and f1-score of the simplified CatBoost models for the 5 definitions of IDH, respectively. ‘Defn1’, ‘Defn2’, ‘Defn3’, ‘Defn4’, and ‘Defn5’ represent the 5 definitions of IDH, respectively. ‘3f’, ‘7f’, ‘8f’, and ‘11f’ represent the simplified CatBoost models with 3 features, 7 features, 8 features, and 11 features, respectively. ‘44f’ represents the original CatBoost model (with 44 features). ROC, Receiver Operating Characteristic Curve; PR, Precision-Recall Curve; AUC, Area Under Curve.
(TIF)

S7 Fig PR curves of the simplified CatBoost models for the 5 definitions of IDH. A–E show the PR curves and PR-AUC of the simplified CatBoost models for the 5 definitions of IDH, respectively. ‘Defn1’, ‘Defn2’, ‘Defn3’, ‘Defn4’, and ‘Defn5’ represent the 5 definitions of IDH, respectively. ‘3f’, ‘7f’, ‘8f’, and ‘11f’ represent the simplified CatBoost models with 3 features, 7 features, 8 features, and 11 features, respectively. ‘44f’ represents the original CatBoost model (with 44 features). PR, Precision-Recall Curve; AUC, Area Under Curve.
(TIF)

S8 Fig Model validation of the original CatBoost models and the simplified CatBoost models for the 5 definitions of IDH. A and B show the model validation (ROC-AUC, PR-AUC, accuracy, precision, recall, and f1-score) results of the original CatBoost models and the simplified CatBoost models for the 5 definitions of IDH, respectively. ‘Defn1’, ‘Defn2’, ‘Defn3’, ‘Defn4’, and ‘Defn5’ represent the 5 definitions of IDH, respectively. ‘11f’ represents the simplified CatBoost model (with 11 features), and ‘44f’ represents the original CatBoost model (with 44 features). ROC, Receiver Operating Characteristic Curve; PR, Precision-Recall Curve; AUC, Area Under Curve.
(TIF)

S9 Fig Model validation (PR curves) of the original CatBoost models and the simplified CatBoost models for the 5 definitions of IDH. A and B show the model validation (PR curves and PR-AUC) results of the original CatBoost models and the simplified CatBoost models for the 5 definitions of IDH, respectively. ‘Defn1’, ‘Defn2’, ‘Defn3’, ‘Defn4’, and ‘Defn5’ represent the 5 definitions of IDH, respectively. ‘11f’ represents the simplified CatBoost model (with 11 features), and ‘44f’ represents the original CatBoost model (with 44 features). PR, Precision-Recall Curve; AUC, Area Under Curve.
(TIF)

S10 Fig Model validation (ROC curves) of the CatBoost model using 15 newly recruited patients. A and B show the model validation (ROC curves and ROC-AUC) results of the original CatBoost models and the simplified CatBoost models for the 5 definitions of IDH, respectively. ‘Defn1’, ‘Defn2’, ‘Defn3’, ‘Defn4’, and ‘Defn5’ represent the 5 definitions of IDH, respectively. ROC, Receiver Operating Characteristic Curve; AUC, Area Under Curve.
(TIF)

S11 Fig ROC curve and PR curve of the machine learning model for the overlapping definitions. ROC, Receiver Operating Characteristic Curve; PR, Precision-Recall Curve; AUC, Area Under Curve; Defn_OL: overlapping definitions.
(TIF)

S12 Fig An IDH prediction example of a standardized patient. A shows the input of feature values for a simulated standardized patient; B–F show the predicted probabilities of IDH and corresponding SHAP plots for definitions 1–5, respectively. G shows the predicted probability of IDH and corresponding SHAP plots for overlapping definitions.
(TIF)

S1 Table ROC-AUC of 10 machine learning models for the 5 definitions of IDH. Results are shown for the ROC-AUC of the 10 machine learning algorithm models for the 5 definitions of IDH, with their 95% confidence intervals shown in parentheses. ROC, Receiver Operating Characteristic Curve; AUC, Area Under Curve. KNN, k-nearest neighbor; LR, Logistic Regression; DT, Decision Tree; ET, Extremely randomized Tree; RF, Random Forest; GBDT, Gradient Boosting Decision Tree; LGBM, Light Gradient Boosting Machine; XGBoost, Extreme Gradient Boosting; AdaBoost, Adaptive Boosting. ‘Defn1’, ‘Defn2’, ‘Defn3’, ‘Defn4’, and ‘Defn5’ represent the 5 definitions of IDH, respectively.
(PDF)

S2 Table Summary of importance of features for the 5 definitions of IDH. Results are shown for the Summary of the importance of features for the 5 definitions of IDH. ‘√√√√’, ‘√√√’, ‘√√’, and ‘√’ represent the features in the top 5, top 10, top 15, and top 20 in order of importance, respectively. ‘Defn1’, ‘Defn2’, ‘Defn3’, ‘Defn4’, and ‘Defn5’ represent the 5 definitions of IDH, respectively. ‘All’ represents the features in the top 5, top 10, top 15, or top 20 in the order of importance for all definitions of IDH.
(PDF)

S3 Table SHAP values and importance ranks of LVMI across blood pressure strata for the 5 definitions of IDH. Results are shown for the SHAP values and importance ranks of LVMI across blood pressure strata for the 5 definitions of IDH. ‘Defn1’, ‘Defn2’, ‘Defn3’, ‘Defn4’, and ‘Defn5’ represent the 5 definitions of IDH, respectively.
(PDF)

S4 Table ROC-AUC of simplified machine learning models for the 5 definitions of IDH. Results are shown for the ROC-AUC of the simplified machine learning models for the 5 definitions of IDH, with their 95% confidence intervals shown in parentheses. ROC, Receiver Operating Characteristic Curve; AUC, Area Under Curve. ‘3 features’, ‘7 features’, ‘8 features’, and ‘11 features’ represent the machine learning models built by simplifying the number of features to 3, 7, 8, and 11, respectively. ‘44 features’ represents the original machine learning model (with 44 features). ‘Defn1’, ‘Defn2’, ‘Defn3’, ‘Defn4’, and ‘Defn5’ represent the 5 definitions of IDH, respectively.
(PDF)

S5 Table ROC-AUC of temporal validation for the 5 definitions of IDH. Results are shown for the ROC-AUC of the temporal validation for the 5 definitions of IDH, with their 95% confidence intervals shown in parentheses. ROC, Receiver Operating Characteristic Curve; AUC, Area Under Curve. ‘valid-44f’ and ‘valid-11f’ represent the original and the simplified machine learning model, respectively. ‘Defn1’, ‘Defn2’, ‘Defn3’, ‘Defn4’, and ‘Defn5’ represent the 5 definitions of IDH, respectively.
(PDF)

S6 Table Model evaluation of the machine learning model for the overlapping definitions. Results are shown for the model evaluation (ROC-AUC, PR-AUC, accuracy, precision, recall, and f1-score) results of the machine learning model for the overlapping definitions. ROC, Receiver Operating Characteristic Curve; AUC, Area Under Curve; CI, confidence intervals.
(PDF)

## References

[1] Bikbov B, Purcell CA, Levey AS, Smith M, Abdoli A, Abebe M, et al. Global, regional, and national burden of chronic kidney disease, 1990–2017: a systematic analysis for the Global Burden of Disease Study 2017. Lancet. 2020;395(10225):709–33. doi: 10.1016/s0140-6736(20)30045-3 32061315 PMC7049905

[2] Himmelfarb J, Ikizler TA. Hemodialysis. N Engl J Med. 2010;363(19):1833–45. doi: 10.1056/NEJMra0902710 21047227

[3] Liyanage T, Ninomiya T, Jha V, Neal B, Patrice HM, Okpechi I, et al. Worldwide access to treatment for end-stage kidney disease: a systematic review. Lancet. 2015;385(9981):1975–82. doi: 10.1016/S0140-6736(14)61601-9 25777665

[4] Keane DF, Raimann JG, Zhang H, Willetts J, Thijssen S, Kotanko P. The time of onset of intradialytic hypotension during a hemodialysis session associates with clinical parameters and mortality. Kidney Int. 2021;99(6):1408–17. doi: 10.1016/j.kint.2021.01.018 33607178 PMC8165353

[5] Reeves PB, Mc Causland FR. Mechanisms, Clinical Implications, and Treatment of Intradialytic Hypotension. Clin J Am Soc Nephrol. 2018;13(8):1297–303. doi: 10.2215/CJN.12141017 29483138 PMC6086712

[6] He J, Baxter SL, Xu J, Xu J, Zhou X, Zhang K. The practical implementation of artificial intelligence technologies in medicine. Nat Med. 2019;25(1):30–6. doi: 10.1038/s41591-018-0307-0 30617336 PMC6995276

[7] Johnson KW, Torres Soto J, Glicksberg BS, Shameer K, Miotto R, Ali M, et al. Artificial Intelligence in Cardiology. J Am Coll Cardiol. 2018;71(23):2668–79. doi: 10.1016/j.jacc.2018.03.521 29880128

[8] Cammarota G, Ianiro G, Ahern A, Carbone C, Temko A, Claesson MJ, et al. Gut microbiome, big data and machine learning to promote precision medicine for cancer. Nat Rev Gastroenterol Hepatol. 2020;17(10):635–48. doi: 10.1038/s41575-020-0327-3 32647386

[9] Kim JI, Maguire F, Tsang KK, Gouliouris T, Peacock SJ, McAllister TA, et al. Machine Learning for Antimicrobial Resistance Prediction: Current Practice, Limitations, and Clinical Perspective. Clin Microbiol Rev. 2022;35(3):e0017921. doi: 10.1128/cmr.00179-21 35612324 PMC9491192

[10] Rajkomar A, Dean J, Kohane I. Machine Learning in Medicine. N Engl J Med. 2019;380(14):1347–58. doi: 10.1056/NEJMra1814259 30943338

[11] Assimon MM, Flythe JE. Definitions of intradialytic hypotension. Semin Dial. 2017;30(6):464–72. doi: 10.1111/sdi.12626 28691195 PMC5668149

[12] Intradialytic Hypotension Prevention and Treatment Expert Working Group, Renal and Blood Purification Committee, Chinese Medicine Education Society. Expert consensus on the prevention and treatment of intradialytic hypotension (2022). Zhonghua Nei Ke Za Zhi. 2022;61(3):269–81. doi: 10.3760/cma.j.cn112138-20210601-00384 35263968

[13] Huang J-C, Tsai Y-C, Wu P-Y, Lien Y-H, Chien C-Y, Kuo C-F, et al. Predictive modeling of blood pressure during hemodialysis: a comparison of linear model, random forest, support vector regression, XGBoost, LASSO regression and ensemble method. Comput Methods Programs Biomed. 2020;195:105536. doi: 10.1016/j.cmpb.2020.105536 32485511

[14] Lin C-J, Chen C-Y, Wu P-C, Pan C-F, Shih H-M, Huang M-Y, et al. Intelligent system to predict intradialytic hypotension in chronic hemodialysis. J Formos Med Assoc. 2018;117(10):888–93. doi: 10.1016/j.jfma.2018.05.023 29941330

[15] Hong D, Chang H, He X, Zhan Y, Tong R, Wu X, et al. Construction of an Early Alert System for Intradialytic Hypotension before Initiating Hemodialysis Based on Machine Learning. Kidney Dis (Basel). 2023;9(5):433–42. doi: 10.1159/000531619 37901708 PMC10601920

[16] Dong J, Wang K, He J, Guo Q, Min H, Tang D, et al. Machine learning-based intradialytic hypotension prediction of patients undergoing hemodialysis: A multicenter retrospective study. Comput Methods Programs Biomed. 2023;240:107698. doi: 10.1016/j.cmpb.2023.107698 37429246

[17] Poorzand H, Sharifipour F, Kerachian A, Ghaderi F, Alimi H, Bigdelu L, et al. Echocardiographic parameters in patients with and without hypotension during dialysis. J Cardiovasc Thorac Res. 2021;13(3):228–33. doi: 10.34172/jcvtr.2021.41 34630971 PMC8493227

[18] Usui N, Nakata J, Uehata A, Ando S, Saitoh M, Kojima S, et al. Association of cardiac autonomic neuropathy assessed by heart rate response during exercise with intradialytic hypotension and mortality in hemodialysis patients. Kidney Int. 2022;101(5):1054–62. doi: 10.1016/j.kint.2022.01.032 35227686

[19] Ozen N, Cepken T. Intradialytic hypotension prevalence, influencing factors, and nursing interventions: prospective results of 744 hemodialysis sessions. Ir J Med Sci. 2020;189(4):1471–6. doi: 10.1007/s11845-020-02249-9 32447597

[20] Agrawal S, Ramachandran P, Gill R, Spitalewitz S, Gunzler D, Silver MR, et al. Erythrocytosis is associated with intradialytic hypotension: a case series. BMC Nephrol. 2019;20(1):235. doi: 10.1186/s12882-019-1426-7 31266452 PMC6604273

[21] Pinter J, Smyth B, Stuard S, Jardine M, Wanner C, Rossignol P, et al. Effect of Dialysate and Plasma Sodium on Mortality in a Global Historical Hemodialysis Cohort. J Am Soc Nephrol. 2023;35(2):167–76. doi: 10.1681/asn.0000000000000262 37967469 PMC10843362

[22] Yang KH, Cho S, Kim SR, Lee Y-J. Serum Phosphorus Levels are Associated with Intradialytic Hypotension in Hemodialysis Patients. Nephron. 2021;145(3):238–44. doi: 10.1159/000513525 33662953

[23] Macedo E, Karl B, Lee E, Mehta RL. A randomized trial of albumin infusion to prevent intradialytic hypotension in hospitalized hypoalbuminemic patients. Crit Care. 2021;25(1):18. doi: 10.1186/s13054-020-03441-0 33407747 PMC7789619

[24] Yang X, Zhao D, Yu F, Heidari AA, Bano Y, Ibrohimov A, et al. An optimized machine learning framework for predicting intradialytic hypotension using indexes of chronic kidney disease-mineral and bone disorders. Comput Biol Med. 2022;145:105510. doi: 10.1016/j.compbiomed.2022.105510 35585728

[25] Lee H, Yun D, Yoo J, Yoo K, Kim YC, Kim DK, et al. Deep Learning Model for Real-Time Prediction of Intradialytic Hypotension. Clin J Am Soc Nephrol. 2021;16(3):396–406. doi: 10.2215/CJN.09280620 33574056 PMC8011016

[26] Kim HW, Heo S-J, Kim M, Lee J, Park KH, Lee G, et al. Deep Learning Model for Predicting Intradialytic Hypotension Without Privacy Infringement: A Retrospective Two-Center Study. Front Med (Lausanne). 2022;9:878858. doi: 10.3389/fmed.2022.878858 35872786 PMC9300869

[27] Gómez-Pulido JA, Gómez-Pulido JM, Rodríguez-Puyol D, Polo-Luque M-L, Vargas-Lombardo M. Predicting the Appearance of Hypotension during Hemodialysis Sessions Using Machine Learning Classifiers. Int J Environ Res Public Health. 2021;18(5):2364. doi: 10.3390/ijerph18052364 33671029 PMC7967733

[28] Othman M, Elbasha AM, Naga YS, Moussa ND. Early prediction of hemodialysis complications employing ensemble techniques. Biomed Eng Online. 2022;21(1):74. doi: 10.1186/s12938-022-01044-0 36221077 PMC9552449

[29] K/DOQI Workgroup. K/DOQI clinical practice guidelines for cardiovascular disease in dialysis patients. Am J Kidney Dis. 2005;45(4 Suppl 3):S1–153. doi: 10.1053/j.ajkd.2005.01.019 15806502

[30] Kooman J, Basci A, Pizzarelli F, Canaud B, Haage P, Fouque D, et al. EBPG guideline on haemodynamic instability. Nephrol Dial Transplant. 2007;22 Suppl 2:ii22–44. doi: 10.1093/ndt/gfm019 17507425

[31] Hirakata H, Nitta K, Inaba M, Shoji T, Fujii H, Kobayashi S, et al, Japanese Society for Dialysis Therapy. Japanese Society for Dialysis Therapy Guidelines for Management of Cardiovascular Diseases in Patients on Chronic Hemodialysis. Ther Apher Dial. 2012;16(5):387–435. doi: 10.1111/j.1744-9987.2012.01088.x 23046367

[32] N Ngankem LSQ, Larizza C, Nocera A, Rombolà G, Quaglini S, Bellazzi R, et al. A comparative study of the definitions of intradialytic hypotension correlated with increased mortality to identify universal predictors. Int J Med Inform. 2023;173:104975. doi: 10.1016/j.ijmedinf.2022.104975 36905746

[33] Yu J, Chen X, Li Y, Wang Y, Liu Z, Shen B, et al. High ultrafiltration rate induced intradialytic hypotension is a predictor for cardiac remodeling: a 5-year cohort study. Ren Fail. 2020;43(1):40–8. doi: 10.1080/0886022x.2020.1853570 PMC7745843 33307918

[34] Hancock JT, Khoshgoftaar TM. CatBoost for big data: an interdisciplinary review. J Big Data. 2020;7(1):94. doi: 10.1186/s40537-020-00369-8 33169094 PMC7610170

[35] Zhang H, Wang L-C, Chaudhuri S, Pickering A, Usvyat L, Larkin J, et al. Real-time prediction of intradialytic hypotension using machine learning and cloud computing infrastructure. Nephrol Dial Transplant. 2023;38(7):1761–9. doi: 10.1093/ndt/gfad070 37055366 PMC10310501

[36] Yoshimura M, Shiramoto H, Koga M, Morimoto Y. Preoperative echocardiography predictive analytics for postinduction hypotension prediction. PLoS ONE. 2022;17(11):e0278140. doi: 10.1371/journal.pone.0278140 PMC9704611 36441797

[37] da Hora Passos R, Caldas J, Ramos JGR, Dos Santos Galvão de Melo EB, Ribeiro MPD, Alves MFC, et al. Ultrasound-based clinical profiles for predicting the risk of intradialytic hypotension in critically ill patients on intermittent dialysis: a prospective observational study. Crit Care. 2019;23(1):389. doi: 10.1186/s13054-019-2668-2 31791373 PMC6889608

