# Supplementary figures and images for: Construction and application of machine learning models for predicting intradialytic hypotension
Source: PLoS One. 2025 Oct 8;20(10):e0333357. doi: 10.1371/journal.pone.0333357 (PMC12507235; doi:10.1371/journal.pone.0333357)

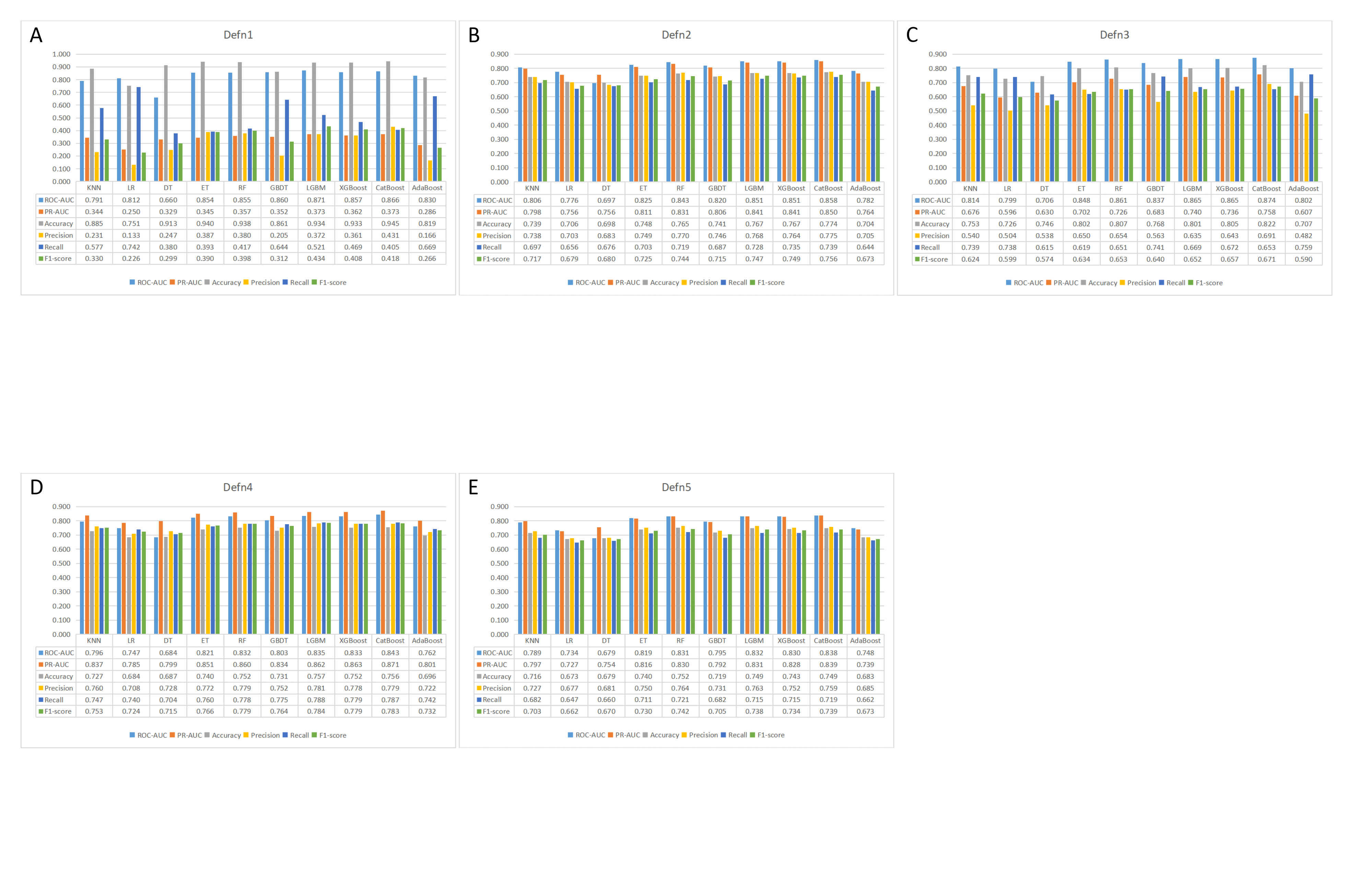

Supplement: S1 Fig — A–E show the ROC-AUC, PR-AUC, accuracy, precision, recall, and f1-score of the 10 machine learning algorithms for the 5 definitions of IDH, respectively. ‘Defn1’, ‘Defn2’, ‘Defn3’, ‘Defn4’, and ‘Defn5’ represent the 5 definitions of IDH, respectively. KNN, k-nearest neighbor; LR, Logistic Regression; DT, Decision Tree; ET, Extremely randomized Tree; RF, Random Forest; GBDT, Gradient Boosting Decision Tree; LGBM, Light Gradient Boosting Machine; XGBoost, Extreme Gradient Boosting; AdaBoost, Adaptive Boosting; ROC, Receiver Operating Characteristic Curve; PR, Precision-Recall Curve; AUC, Area Under Curve. (TIF) [file pone.0333357.s001.tif]

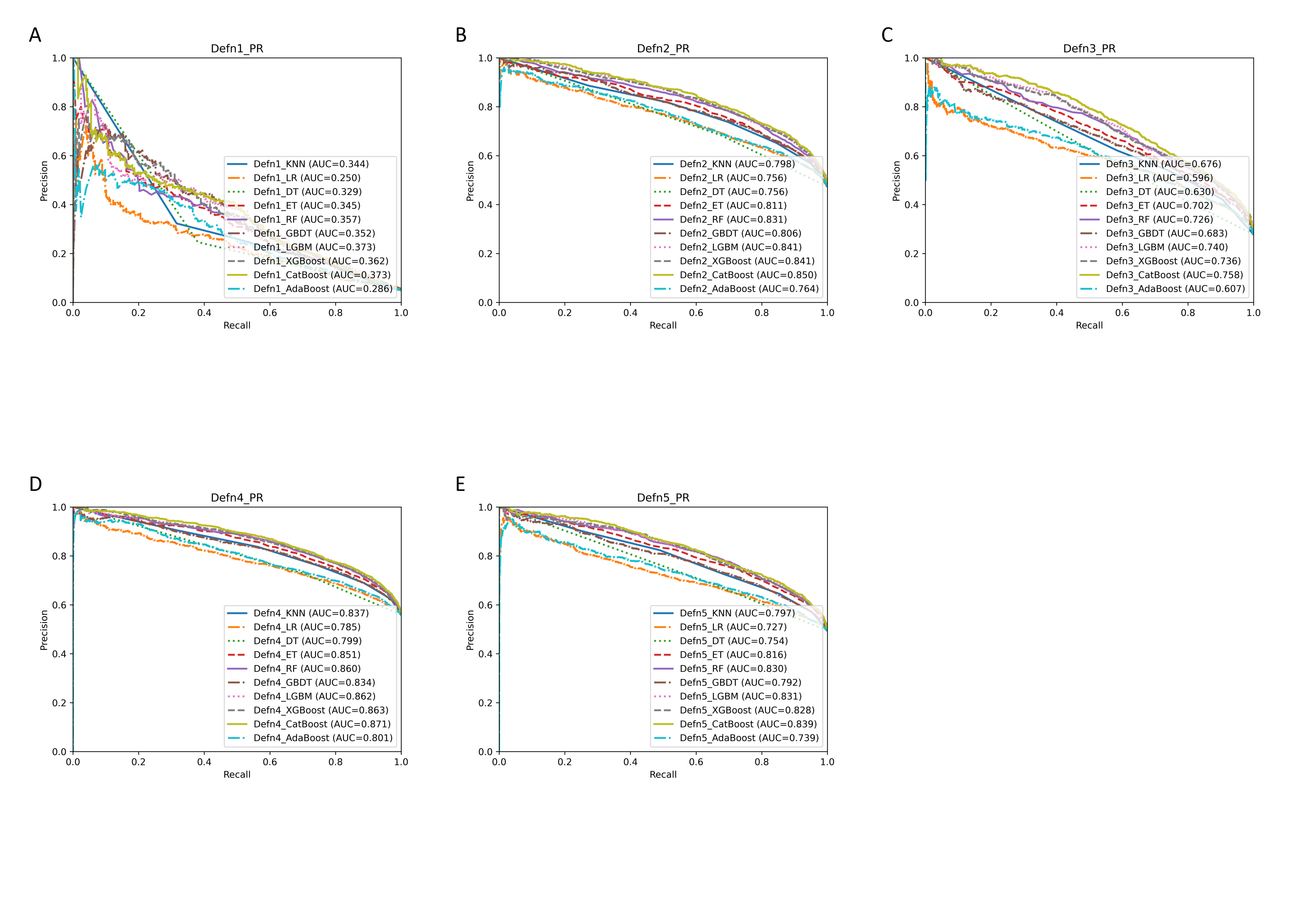

Supplement: S2 Fig — A–E show the PR curves and PR-AUC of 10 machine learning models for the 5 definitions of IDH, respectively. ‘Defn1’, ‘Defn2’, ‘Defn3’, ‘Defn4’, and ‘Defn5’ represent the 5 definitions of IDH, respectively. KNN, k-nearest neighbor; LR, Logistic Regression; DT, Decision Tree; ET, Extremely randomized Tree; RF, Random Forest; GBDT, Gradient Boosting Decision Tree; LGBM, Light Gradient Boosting Machine; XGBoost, Extreme Gradient Boosting; AdaBoost, Adaptive Boosting; PR, Precision-Recall Curve; AUC, Area Under Curve. (TIF) [file pone.0333357.s002.tif]

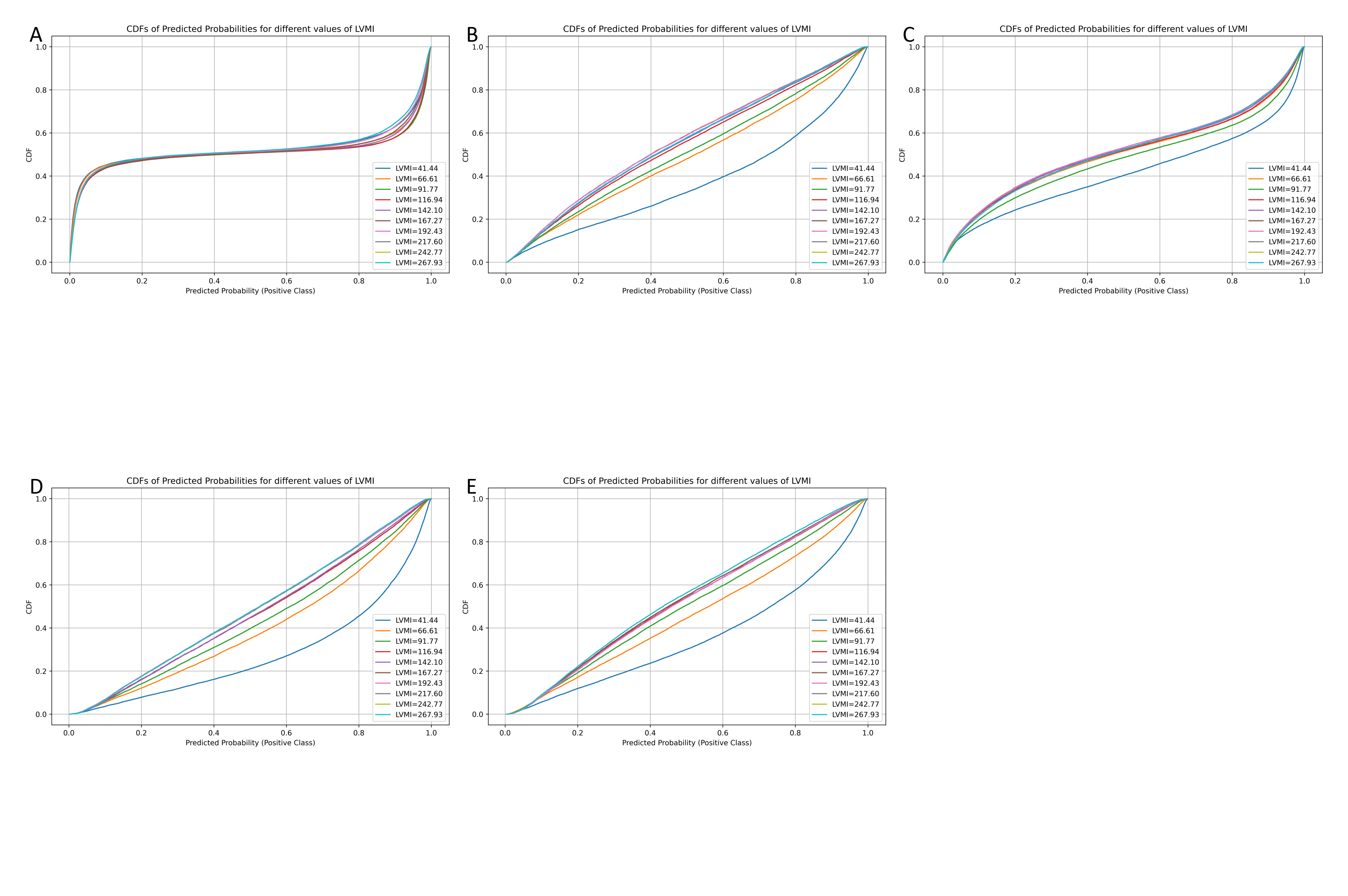

Supplement: S3 Fig — A–E show the CDF curves of the CatBoost model for the 5 definitions of IDH, respectively, with LVMI fixed at one of ten representative values uniformly sampled between its maximum and minimum observed values. CDF, Cumulative Distribution Function; LVMI, Left Ventricular Mass Index. (TIF) [file pone.0333357.s003.tif]

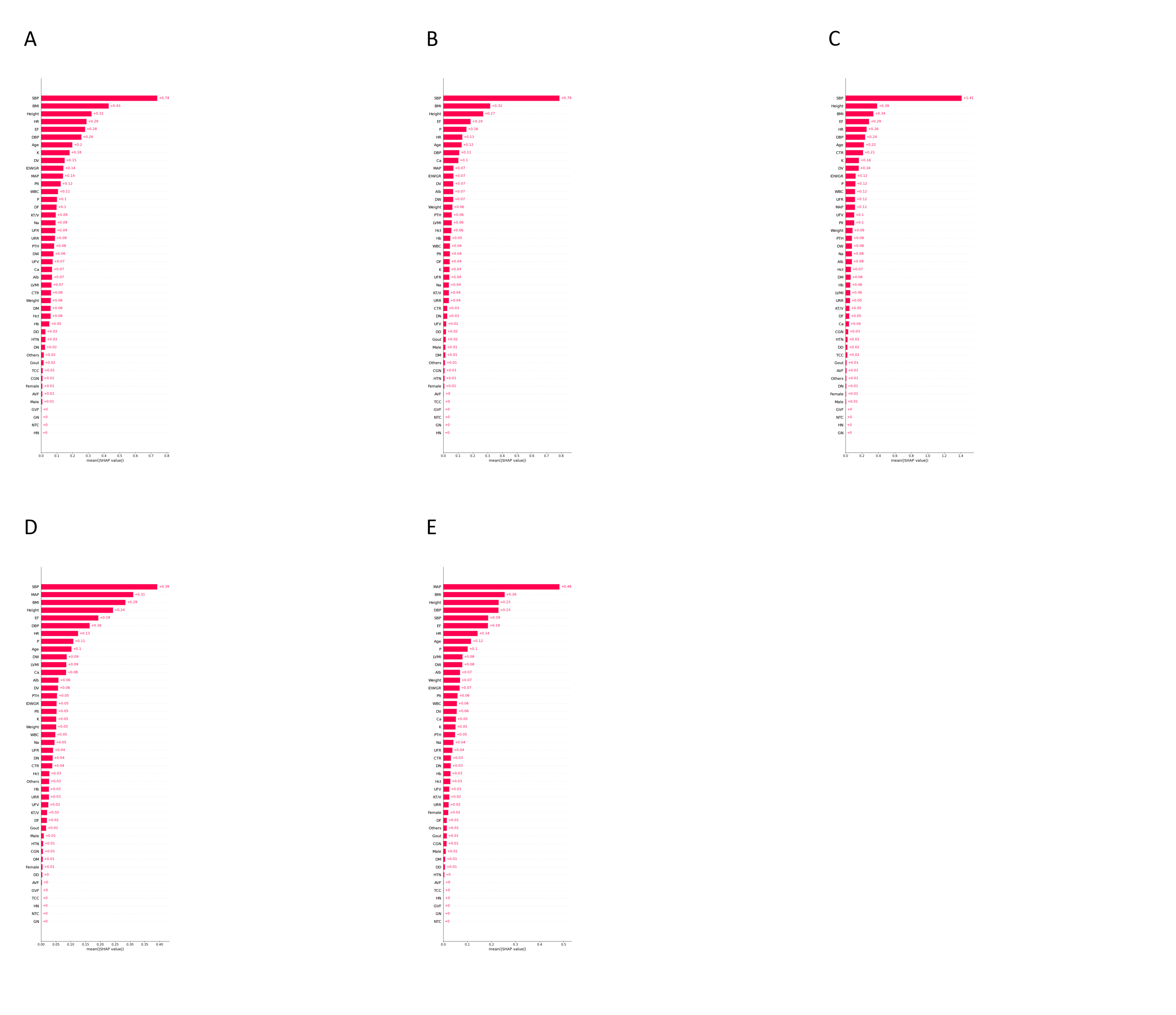

Supplement: S4 Fig — A–E show the SHAP summary plots of CatBoost models for the 5 definitions of IDH in this subgroup, respectively. All features are presented in descending order of importance, with their corresponding SHAP values displayed along the horizontal axis. (TIF) [file pone.0333357.s004.tif]

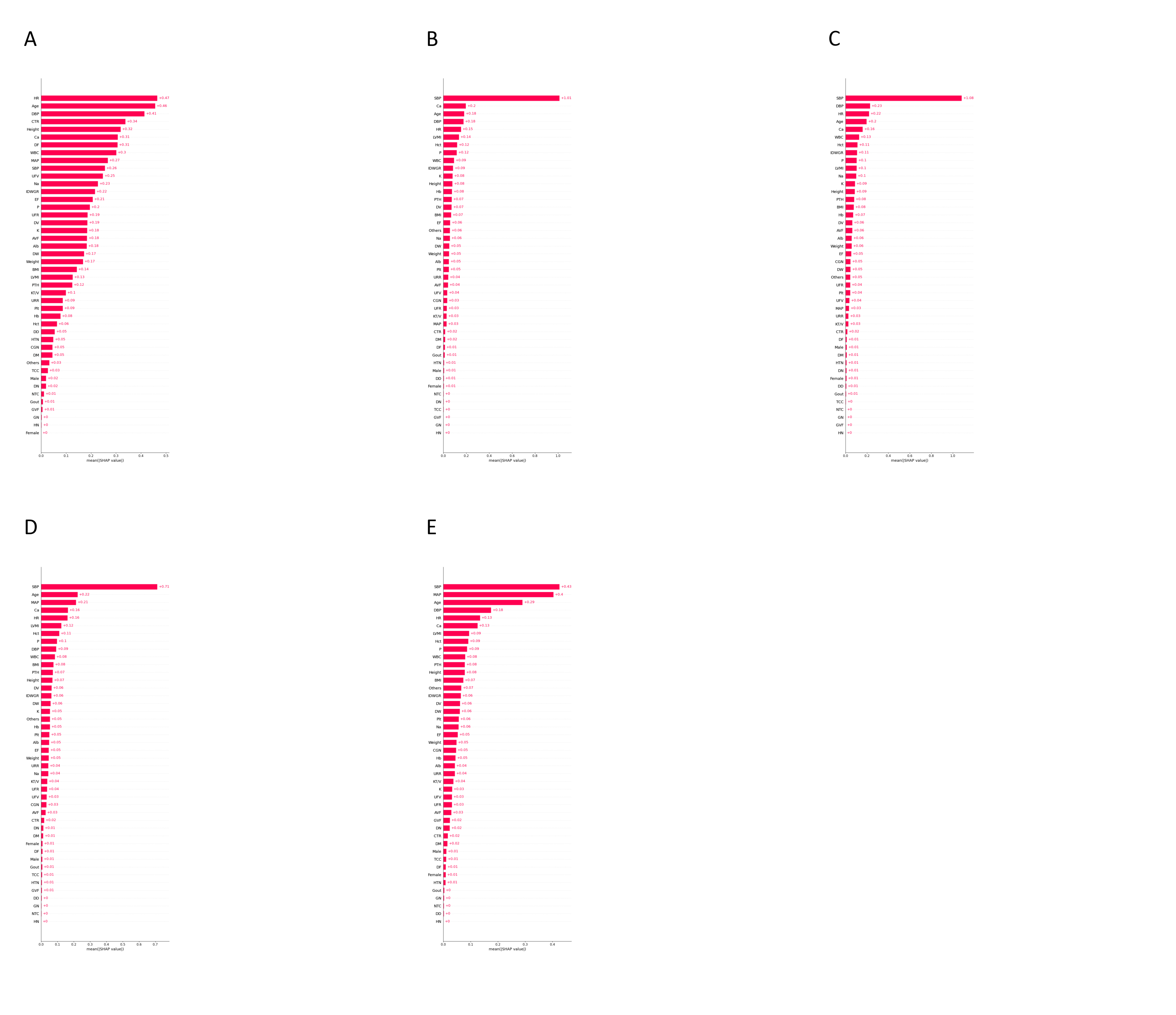

Supplement: S5 Fig — A–E show the SHAP summary plots of CatBoost models for the 5 definitions of IDH in this subgroup, respectively. All features are presented in descending order of importance, with their corresponding SHAP values displayed along the horizontal axis. (TIF) [file pone.0333357.s005.tif]

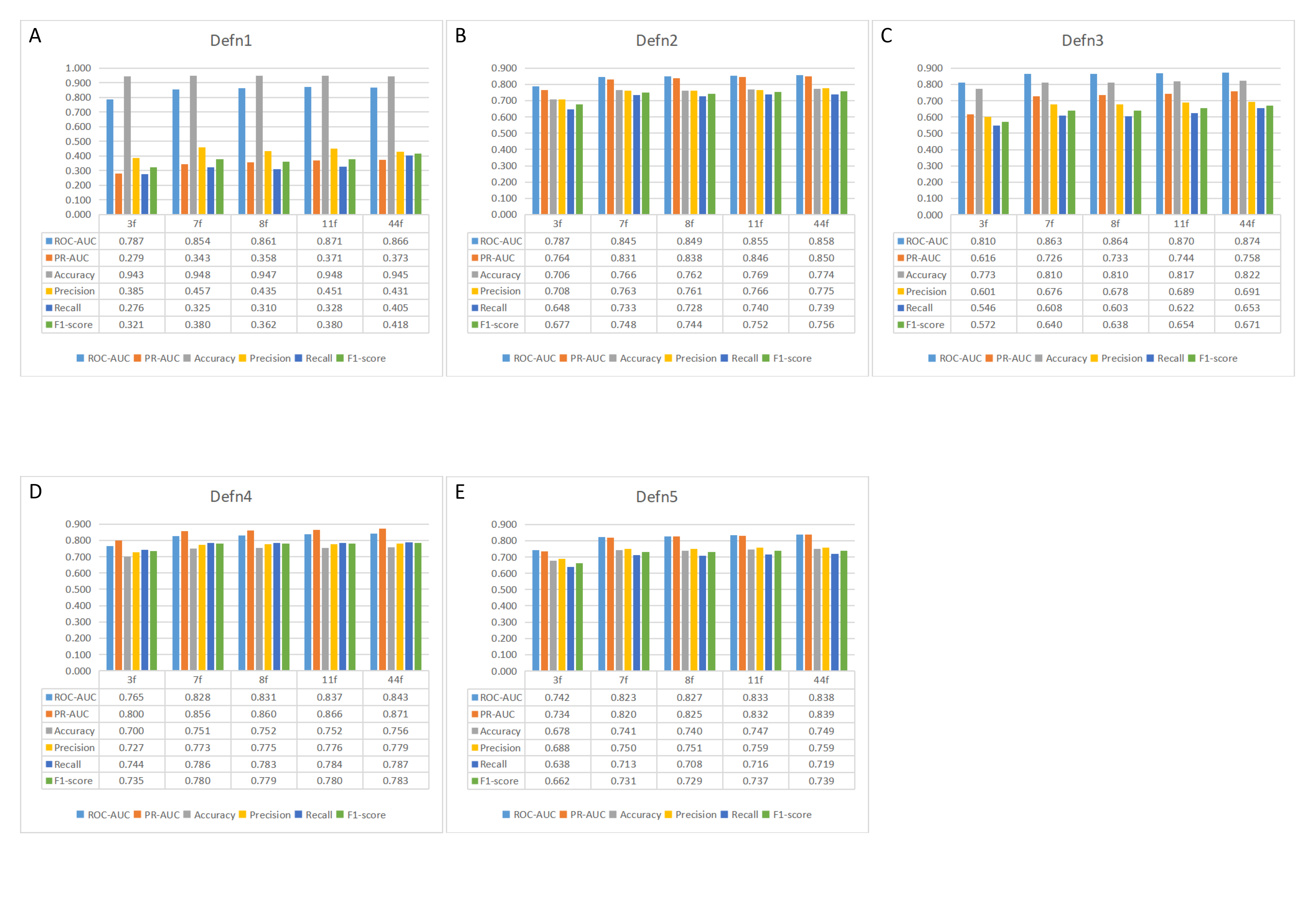

Supplement: S6 Fig — A–E show the ROC-AUC, PR-AUC, accuracy, precision, recall, and f1-score of the simplified CatBoost models for the 5 definitions of IDH, respectively. ‘Defn1’, ‘Defn2’, ‘Defn3’, ‘Defn4’, and ‘Defn5’ represent the 5 definitions of IDH, respectively. ‘3f’, ‘7f’, ‘8f’, and ‘11f’ represent the simplified CatBoost models with 3 features, 7 features, 8 features, and 11 features, respectively. ‘44f’ represents the original CatBoost model (with 44 features). ROC, Receiver Operating Characteristic Curve; PR, Precision-Recall Curve; AUC, Area Under Curve. (TIF) [file pone.0333357.s006.tif]

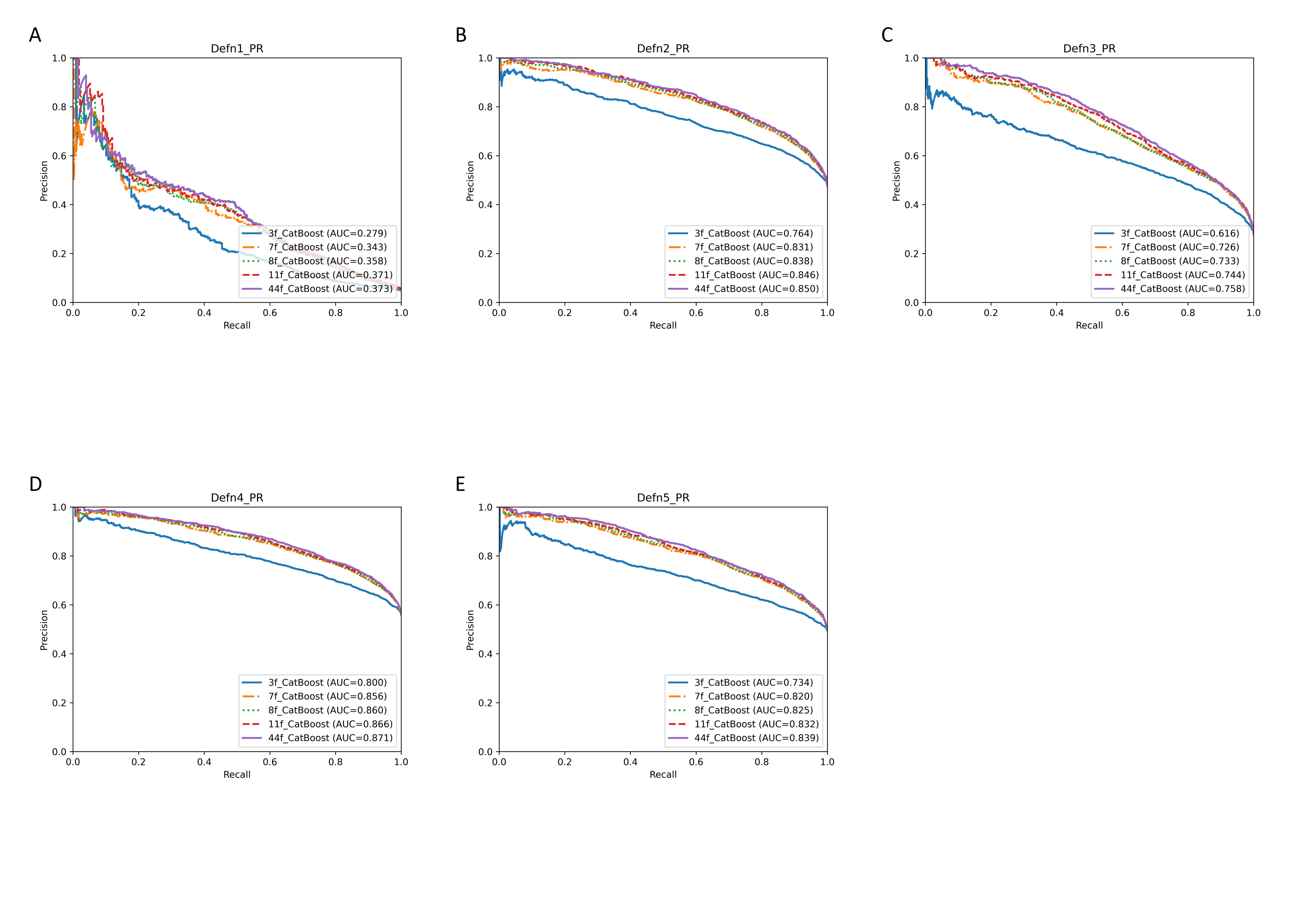

Supplement: S7 Fig — A–E show the PR curves and PR-AUC of the simplified CatBoost models for the 5 definitions of IDH, respectively. ‘Defn1’, ‘Defn2’, ‘Defn3’, ‘Defn4’, and ‘Defn5’ represent the 5 definitions of IDH, respectively. ‘3f’, ‘7f’, ‘8f’, and ‘11f’ represent the simplified CatBoost models with 3 features, 7 features, 8 features, and 11 features, respectively. ‘44f’ represents the original CatBoost model (with 44 features). PR, Precision-Recall Curve; AUC, Area Under Curve. (TIF) [file pone.0333357.s007.tif]

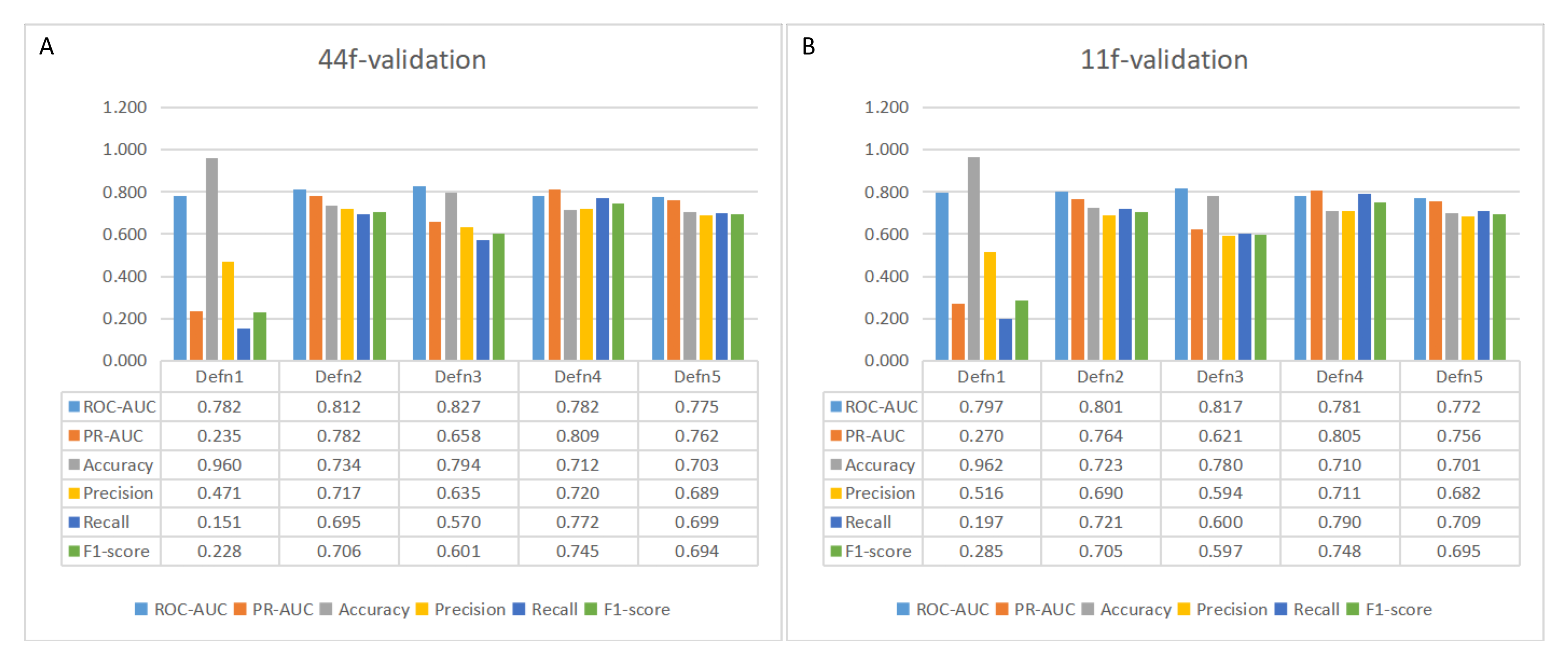

Supplement: S8 Fig — A and B show the model validation (ROC-AUC, PR-AUC, accuracy, precision, recall, and f1-score) results of the original CatBoost models and the simplified CatBoost models for the 5 definitions of IDH, respectively. ‘Defn1’, ‘Defn2’, ‘Defn3’, ‘Defn4’, and ‘Defn5’ represent the 5 definitions of IDH, respectively. ‘11f’ represents the simplified CatBoost model (with 11 features), and ‘44f’ represents the original CatBoost model (with 44 features). ROC, Receiver Operating Characteristic Curve; PR, Precision-Recall Curve; AUC, Area Under Curve. (TIF) [file pone.0333357.s008.tif]

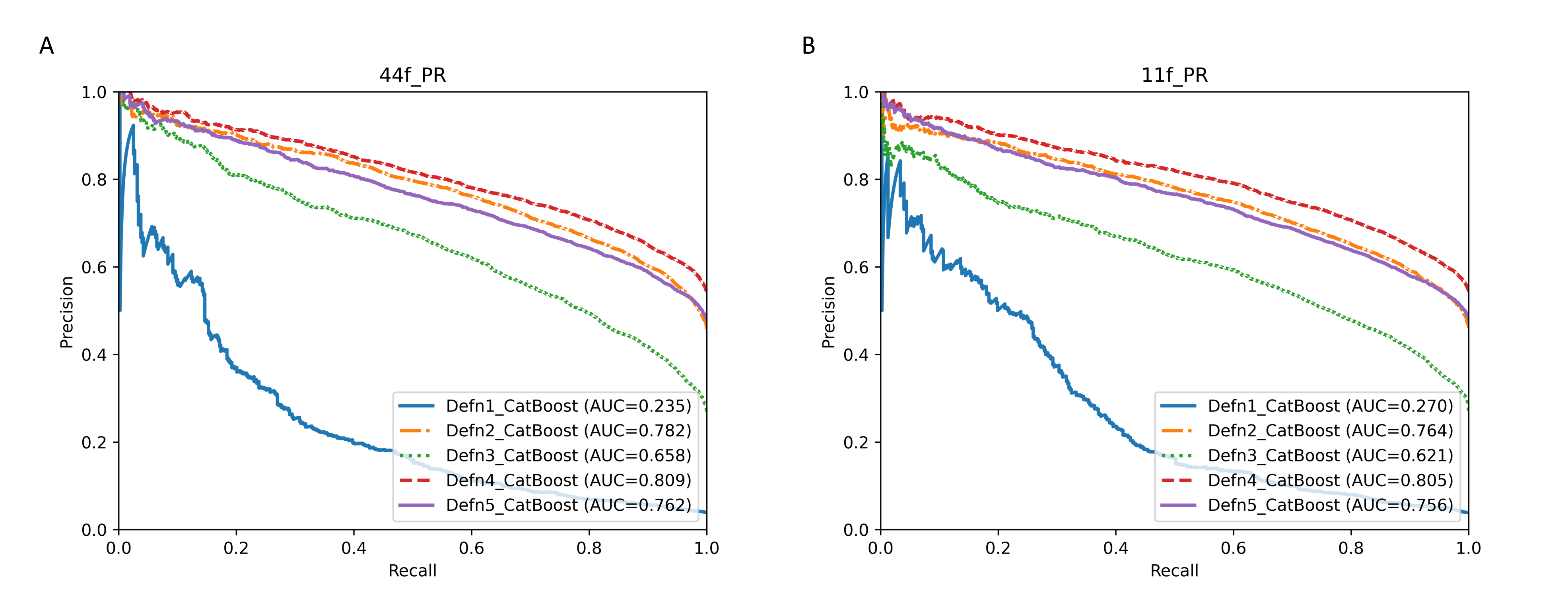

Supplement: S9 Fig — A and B show the model validation (PR curves and PR-AUC) results of the original CatBoost models and the simplified CatBoost models for the 5 definitions of IDH, respectively. ‘Defn1’, ‘Defn2’, ‘Defn3’, ‘Defn4’, and ‘Defn5’ represent the 5 definitions of IDH, respectively. ‘11f’ represents the simplified CatBoost model (with 11 features), and ‘44f’ represents the original CatBoost model (with 44 features). PR, Precision-Recall Curve; AUC, Area Under Curve. (TIF) [file pone.0333357.s009.tif]

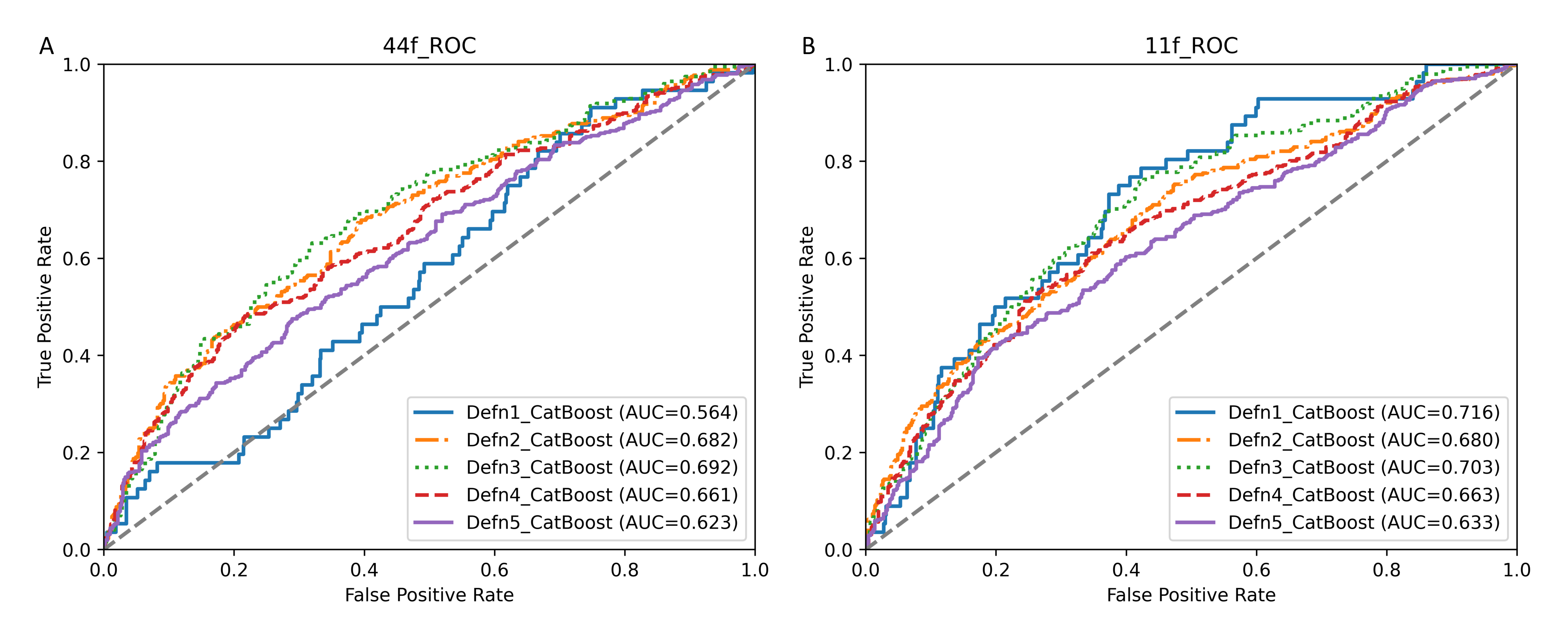

Supplement: S10 Fig — A and B show the model validation (ROC curves and ROC-AUC) results of the original CatBoost models and the simplified CatBoost models for the 5 definitions of IDH, respectively. ‘Defn1’, ‘Defn2’, ‘Defn3’, ‘Defn4’, and ‘Defn5’ represent the 5 definitions of IDH, respectively. ROC, Receiver Operating Characteristic Curve; AUC, Area Under Curve. (TIF) [file pone.0333357.s010.tif]

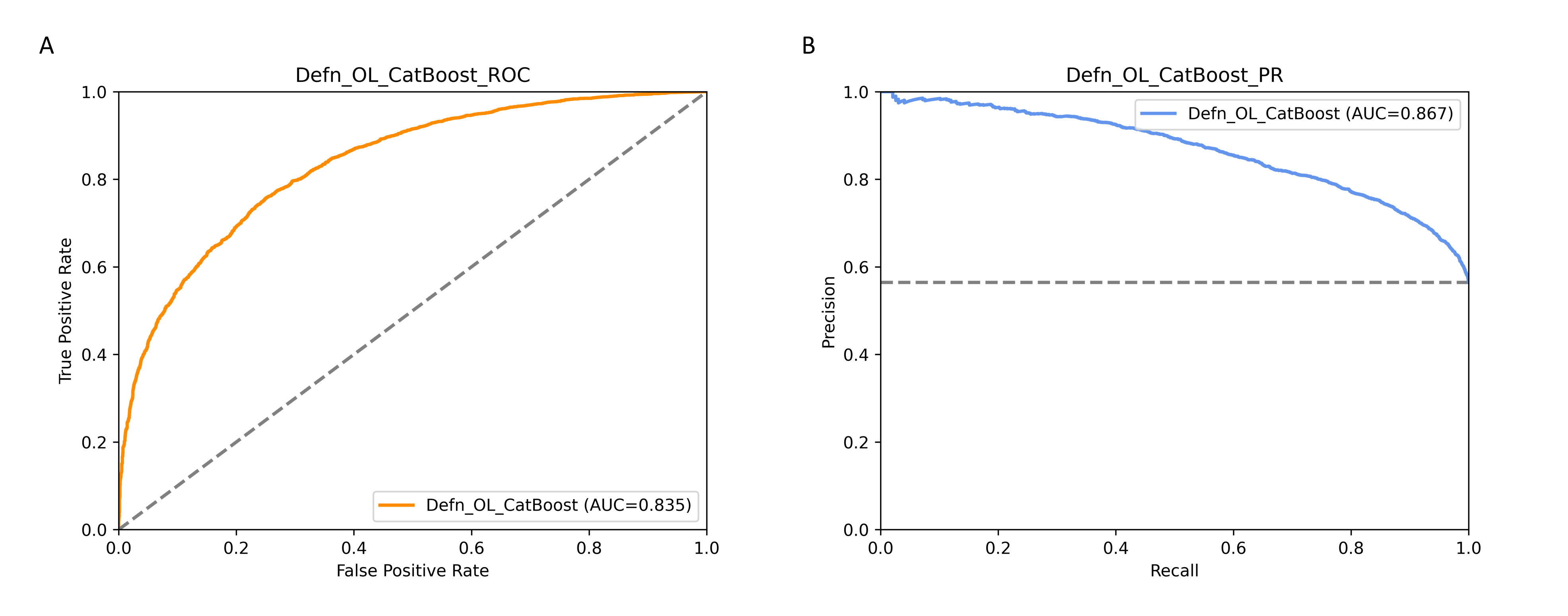

Supplement: S11 Fig — ROC, Receiver Operating Characteristic Curve; PR, Precision-Recall Curve; AUC, Area Under Curve; Defn_OL: overlapping definitions. (TIF) [file pone.0333357.s011.tif]

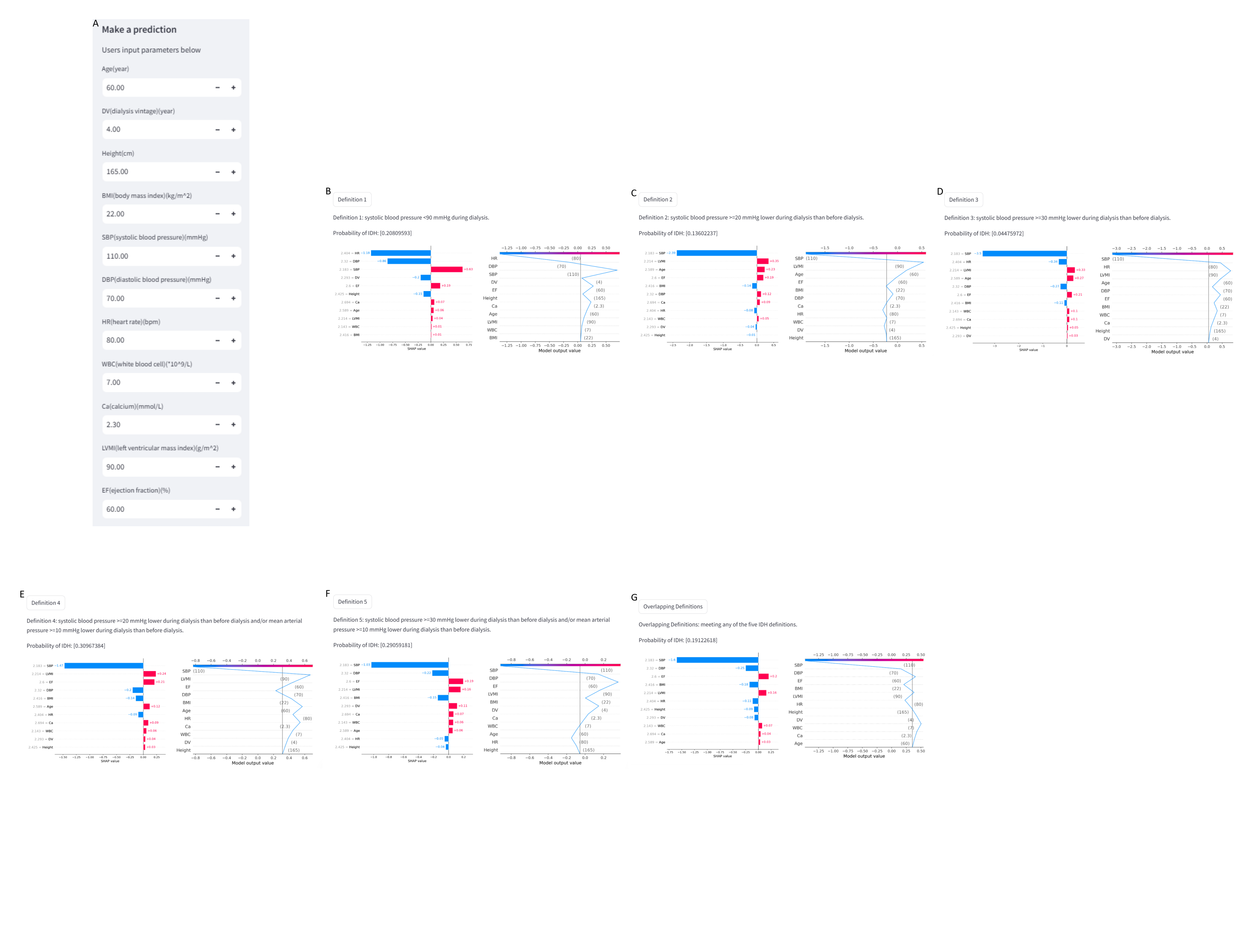

Supplement: S12 Fig — A shows the input of feature values for a simulated standardized patient; B–F show the predicted probabilities of IDH and corresponding SHAP plots for definitions 1–5, respectively. G shows the predicted probability of IDH and corresponding SHAP plots for overlapping definitions. (TIF) [file pone.0333357.s012.tif]
