# Supplementary material for: Construction and application of machine learning models for predicting intradialytic hypotension
Source: PLoS One. 2025 Oct 8;20(10):e0333357. doi: 10.1371/journal.pone.0333357 (PMC12507235; doi:10.1371/journal.pone.0333357)
Supplement: S1 Table — Results are shown for the ROC-AUC of the 10 machine learning algorithm models for the 5 definitions of IDH, with their 95% confidence intervals shown in parentheses. ROC, Receiver Operating Characteristic Curve; AUC, Area Under Curve. KNN, k-nearest neighbor; LR, Logistic Regression; DT, Decision Tree; ET, Extremely randomized Tree; RF, Random Forest; GBDT, Gradient Boosting Decision Tree; LGBM, Light Gradient Boosting Machine; XGBoost, Extreme Gradient Boosting; AdaBoost, Adaptive Boosting. ‘Defn1’, ‘Defn2’, ‘Defn3’, ‘Defn4’, and ‘Defn5’ represent the 5 definitions of IDH, respectively. (PDF) [file pone.0333357.s013.pdf]

S1 Table. ROC-AUC of 10 machine learning models for the 5 definitions of IDH.

	KNN	LR	DT	ET	RF	GBDT	LGBM	XGBoost	CatBoost	AdaBoost
**Defn1**	0.791 (0.762-0.821)	0.812 (0.784-0.841)	0.660 (0.627-0.693)	0.854 (0.828-0.881)	0.855 (0.828-0.881)	0.860 (0.834-0.886)	0.871 (0.846-0.896)	0.857 (0.831-0.883)	0.866 (0.841-0.891)	0.830 (0.802-0.857)
**Defn2**	0.806 (0.796-0.817)	0.776 (0.764-0.787)	0.697 (0.684-0.709)	0.825 (0.815-0.836)	0.843 (0.833-0.852)	0.820 (0.810-0.831)	0.851 (0.841-0.860)	0.851 (0.841-0.860)	0.858 (0.849-0.867)	0.782 (0.771-0.793)
**Defn3**	0.814 (0.802-0.827)	0.799 (0.786-0.812)	0.706 (691-0.721)	0.848 (0.836-0.859)	0.861 (0.850-0.872)	0.837 (0.825-0.849)	0.865 (0.854-0.876)	0.865 (0.854-0.876)	0.874 (0.863-0.885)	0.802 (0.789-0.815)
**Defn4**	0.796 (0.785-0.806)	0.747 (0.735-0.758)	0.684 (0.672-0.697)	0.821 (0.811-0.830)	0.832 (0.822-0.841)	0.803 (0.793-0.814)	0.835 (0.826-0.845)	0.833 (0.823-0.842)	0.843 (0.833-0.852)	0.762 (0.750-0.773)
**Defn5**	0.789 (0.778-0.800)	0.734 (0.722-0.746)	0.679 (0.666-0.692)	0.819 (0.809-0.829)	0.831 (0.821-0.841)	0.795 (0.784-0.805)	0.832 (0.822-0.842)	0.830 (0.820-0.840)	0.838 (0.828-0.847)	0.748 (0.736-0.759)

Results are shown for the ROC-AUC of the 10 machine learning algorithm models for the 5 definitions of IDH, with their 95% confidence intervals shown in parentheses. ROC, Receiver Operating Characteristic Curve; AUC, Area Under Curve. KNN, k-nearest neighbor; LR, Logistic Regression; DT, Decision Tree; ET, Extremely randomized Tree; RF, Random Forest; GBDT, Gradient Boosting Decision Tree; LGBM, Light Gradient Boosting Machine; XGBoost, Extreme Gradient Boosting; AdaBoost, Adaptive Boosting. ‘Defn1’, ‘Defn2’, ‘Defn3’, ‘Defn4’, and ‘Defn5’ represent the 5 definitions of IDH, respectively.
