# Supplementary material for: Construction and application of machine learning models for predicting intradialytic hypotension
Source: PLoS One. 2025 Oct 8;20(10):e0333357. doi: 10.1371/journal.pone.0333357 (PMC12507235; doi:10.1371/journal.pone.0333357)
Supplement: S2 Table — Results are shown for the Summary of the importance of features for the 5 definitions of IDH. ‘√√√√’, ‘√√√’, ‘√√’, and ‘√’ represent the features in the top 5, top 10, top 15, and top 20 in order of importance, respectively. ‘Defn1’, ‘Defn2’, ‘Defn3’, ‘Defn4’, and ‘Defn5’ represent the 5 definitions of IDH, respectively. ‘All’ represents the features in the top 5, top 10, top 15, or top 20 in the order of importance for all definitions of IDH. (PDF) [file pone.0333357.s014.pdf]

S2 Table. Summary of importance of features for the 5 definitions of IDH.

	Defn1	Defn2	Defn3	Defn4	Defn5	All
**Demographic characteristics**						
Male (n;%)						
Female (n;%)						
Age (year)	√√√√	√√√√	√√√√	√√√√	√√√√	√√√√
DV (dialysis vintage) (year)	√√	√√	√	√√	√	√
Height (cm)	√√√	√√√	√√√	√√√	√√√	√√√
Weight (kg)		√	√		√	
BMI (kg/m^2)	√√√	√√√√	√√√	√√√√	√√√	√√√
**Primary diseases**						
HN (hypertensive nephrosclerosis) (n;%)						
DN (diabetic nephropathy) (n;%)						
GN (gouty nephropathy) (n;%)						
CGN (chronic glomerulonephritis) (n;%)						
Others (n;%)				√	√√	
**Comorbidities**						
HTN (hypertension) (n;%)						
DM (diabetes mellitus) (n;%)						
Gout (n;%)						
**Vital signs**						
SBP (systolic blood pressure) (mmHg)	√√√√	√√√√	√√√√	√√√√	√√√√	√√√√
DBP (diastolic blood pressure) (mmHg)	√√√√	√√√	√√√√	√√√	√√√√	√√√
MAP (mean arterial pressure) (mmHg)	√√√√			√√√√	√√√√	
HR (heart rate) (bpm)	√√√√	√√√√	√√√√	√√√√	√√√√	√√√√
**Laboratory tests**						
WBC (white blood cell) (*10^9/L)	√√	√√	√√√	√	√√	√
Hb (hemoglobin) (g/L)	√		√√			
Hct (hematocrit) (%)		√√√	√	√√	√	
Plt (platelet) (*10^9/L)		√	√	√√	√√	
K (potassium) (mmol/L)	√√	√√	√√	√		
Na (sodium) (mmol/L)	√	√	√√		√	
P (phosphorus) (mmol/L)		√√√	√√√	√√√	√√√	
Ca (calcium) (mmol/L)	√√√	√√√√	√√√√	√√√	√√√	√√√
Alb (albumin) (g/L)		√				
PTH (parathyroid hormone) (pg/ml)		√√	√	√√	√√	
**Ultrasound and imaging examinations**						
CTR (cardiothoracic ratio)	√√					
LVMI (left ventricular mass index) (g/m^2)	√	√√√	√√√	√√√	√√√	√
EF (ejection fraction) (%)	√√√	√√	√√	√√	√√	√√
**Hemodialysis access**						
AVF (autologous arteriovenous fistula) (n;%)						
GVF (grafted vascular fistula) (n;%)						
TCC (Tunneled Cuffed Catheter) (n;%)						

**NTC (Non-tunneled Catheter) (n;%)**						
**Dialysis settings**						
**DF (dialysis frequency) (per week)**						
**DD (dialysis duration) (h)**						
**DW (dry weight) (kg)**	√			√	√	
**IDWGR (interdialytic weight gain rate)**	√	√	√√			
**UFV (ultrafiltration volume) (L)**	√√√					
**UFR (ultrafiltration rate) (%)**	√√					
**Dialysis adequacy indexes**						
**Kt/V (urea clearance index)**						
**URR (urea reduction ratio)**				√		

Results are shown for the Summary of the importance of features for the 5 definitions of IDH. '√√√√', '√√√', '√√', and '√' represent the features in the top 5, top 10, top 15, and top 20 in order of importance, respectively. 'Defn1', 'Defn2', 'Defn3', 'Defn4', and 'Defn5' represent the 5 definitions of IDH, respectively. 'All' represents the features in the top 5, top 10, top 15, or top 20 in the order of importance for all definitions of IDH.
