# Supplementary material for: Construction and application of machine learning models for predicting intradialytic hypotension
Source: PLoS One. 2025 Oct 8;20(10):e0333357. doi: 10.1371/journal.pone.0333357 (PMC12507235; doi:10.1371/journal.pone.0333357)
Supplement: S3 Table — Results are shown for the SHAP values and importance ranks of LVMI across blood pressure strata for the 5 definitions of IDH. ‘Defn1’, ‘Defn2’, ‘Defn3’, ‘Defn4’, and ‘Defn5’ represent the 5 definitions of IDH, respectively. (PDF) [file pone.0333357.s015.pdf]

S3 Table. SHAP values and importance ranks of LVMI across blood pressure strata for the 5 definitions of IDH.

	90mmHg≤SBP＜130mmHg subgroup		SBP≥130mmHg subgroup	
	**SHAP value**	**Importance rank**	**SHAP value**	**Importance rank**
**Defn1**	0.07	25	0.13	24
**Defn2**	0.06	17	0.14	6
**Defn3**	0.06	26	0.10	10
**Defn4**	0.09	11	0.12	6
**Defn5**	0.08	10	0.09	7

Results are shown for the SHAP values and importance ranks of LVMI across blood pressure strata for the 5 definitions of IDH. ‘Defn1’, ‘Defn2’, ‘Defn3’, ‘Defn4’, and ‘Defn5’ represent the 5 definitions of IDH, respectively.
