# Supplementary material for: Construction and application of machine learning models for predicting intradialytic hypotension
Source: PLoS One. 2025 Oct 8;20(10):e0333357. doi: 10.1371/journal.pone.0333357 (PMC12507235; doi:10.1371/journal.pone.0333357)
Supplement: S4 Table — Results are shown for the ROC-AUC of the simplified machine learning models for the 5 definitions of IDH, with their 95% confidence intervals shown in parentheses. ROC, Receiver Operating Characteristic Curve; AUC, Area Under Curve. ‘3 features’, ‘7 features’, ‘8 features’, and ‘11 features’ represent the machine learning models built by simplifying the number of features to 3, 7, 8, and 11, respectively. ‘44 features’ represents the original machine learning model (with 44 features). ‘Defn1’, ‘Defn2’, ‘Defn3’, ‘Defn4’, and ‘Defn5’ represent the 5 definitions of IDH, respectively. (PDF) [file pone.0333357.s016.pdf]

S4 Table. ROC-AUC of simplified machine learning models for the 5 definitions of IDH.

	3 features	7 features	8 features	11 features	44 features
**Defn1**	0.787 (0.757-0.817)	0.854 (0.828-0.881)	0.861 (0.835-0.887)	0.871 (0.846-0.896)	0.866 (0.841-0.891)
**Defn2**	0.787 (0.776-0.798)	0.845 (0.835-0.854)	0.849 (0.839-0.858)	0.855 (0.846-0.864)	0.858 (0.849-0.867)
**Defn3**	0.810 (0.798-0.823)	0.863 (0.851-0.874)	0.864 (0.853-0.875)	0.870 (0.859-0.881)	0.874 (0.863-0.885)
**Defn4**	0.765 (0.753-0.776)	0.828 (0.819-0.838)	0.831 (0.821-0.841)	0.837 (0.828-0.846)	0.843 (0.833-0.852)
**Defn5**	0.742 (0.730-0.754)	0.823 (0.813-0.833)	0.827 (0.817-0.837)	0.833 (0.823-0.843)	0.838 (0.828-0.847)

Results are shown for the ROC-AUC of the simplified machine learning models for the 5 definitions of IDH, with their 95% confidence intervals shown in parentheses. ROC, Receiver Operating Characteristic Curve; AUC, Area Under Curve. ‘3 features’, ‘7 features’, ‘8 features’, and ‘11 features’ represent the machine learning models built by simplifying the number of features to 3, 7, 8, and 11, respectively. ‘44 features’ represents the original machine learning model (with 44 features). ‘Defn1’, ‘Defn2’, ‘Defn3’, ‘Defn4’, and ‘Defn5’ represent the 5 definitions of IDH, respectively.
