# Supplementary material for: Construction and application of machine learning models for predicting intradialytic hypotension
Source: PLoS One. 2025 Oct 8;20(10):e0333357. doi: 10.1371/journal.pone.0333357 (PMC12507235; doi:10.1371/journal.pone.0333357)
Supplement: S5 Table — Results are shown for the ROC-AUC of the temporal validation for the 5 definitions of IDH, with their 95% confidence intervals shown in parentheses. ROC, Receiver Operating Characteristic Curve; AUC, Area Under Curve. ‘valid-44f’ and ‘valid-11f’ represent the original and the simplified machine learning model, respectively. ‘Defn1’, ‘Defn2’, ‘Defn3’, ‘Defn4’, and ‘Defn5’ represent the 5 definitions of IDH, respectively. (PDF) [file pone.0333357.s017.pdf]

S5 Table. ROC-AUC of temporal validation for the 5 definitions of IDH.

	valid-44f	valid-11f
**Defn1**	0.782 (0.757-0.807)	0.797 (0.773-0.821)
**Defn2**	0.812 (0.804-0.820)	0.801 (0.793-0.809)
**Defn3**	0.827 (0.818-0.836)	0.817 (0.807-0.826)
**Defn4**	0.782 (0.774-0.790)	0.781 (0.773-0.789)
**Defn5**	0.775 (0.767-0.784)	0.772 (0.764-0.780)

Results are shown for the ROC-AUC of the temporal validation for the 5 definitions of IDH, with their 95% confidence intervals shown in parentheses. ROC, Receiver Operating Characteristic Curve; AUC, Area Under Curve. 'valid-44f' and 'valid-11f' represent the original and the simplified machine learning model, respectively. 'Defn1', 'Defn2', 'Defn3', 'Defn4', and 'Defn5' represent the 5 definitions of IDH, respectively.
