# Supplementary material for: Construction and application of machine learning models for predicting intradialytic hypotension
Source: PLoS One. 2025 Oct 8;20(10):e0333357. doi: 10.1371/journal.pone.0333357 (PMC12507235; doi:10.1371/journal.pone.0333357)
Supplement: S6 Table — Results are shown for the model evaluation (ROC-AUC, PR-AUC, accuracy, precision, recall, and f1-score) results of the machine learning model for the overlapping definitions. ROC, Receiver Operating Characteristic Curve; AUC, Area Under Curve; CI, confidence intervals. (PDF) [file pone.0333357.s018.pdf]

S6 Table. Model evaluation of the machine learning model for the overlapping definitions.

	Model evaluation
**ROC-AUC**	0.835 (95% CI: 0.826-0.845)
**PR-AUC**	0.867
**Accuracy**	0.754
**Precision**	0.778
**Recall**	0.789
**F1-score**	0.784

Results are shown for the model evaluation (ROC-AUC, PR-AUC, accuracy, precision, recall, and f1-score) results of the machine learning model for the overlapping definitions. ROC, Receiver Operating Characteristic Curve; AUC, Area Under Curve; CI, confidence intervals.
